# Microblasting Wound Dressings Mechanically Disrupt Polymicrobial Biofilms to Enhance Healing in Treatment‐Resistant Wounds

**DOI:** 10.1002/advs.75999

**Published:** 2026-06-10

**Authors:** Yujin Ahn, Joo Hun Lee, Christian Hurd, Jiye Lee, Junggeon Park, Adam A. Markowicz, Zheyuan Zhang, Joanne Hwang, Guillermo L. Monroy, Simon A. Rogers, Woonggyu Jung, Stephen A. Boppart, Hyunjoon Kong

**Affiliations:** ^1^ Department of Chemical and Biomolecular Engineering University of Illinois at Urbana‐Champaign Urbana Illinois USA; ^2^ Chan Zuckerberg Biohub Chicago Chicago Illinois USA; ^3^ Department of Bioengineering University of Illinois at Urbana‐Champaign Urbana Illinois USA; ^4^ Beckman Institute for Advanced Science and Technology University of Illinois at Urbana‐Champaign Urbana Illinois USA; ^5^ Deparment of Biomedical Engineering Ulsan National Institute of Science and Technology Ulsan Republic of Korea; ^6^ Department of Electrical and Computer Engineering University of Illinois at Urbana‐Champaign Urbana Illinois USA; ^7^ NIH/NIBIB Center for Label‐free Imaging and Multiscale Biophotonics (CLIMB) University of Illinois at Urbana‐Champaign Urbana Illinois USA; ^8^ Scott H. Fisher Multi‐Cellular Engineered Living Systems Theme Carl R. Woese Institute for Genomic Biology University of Illinois at Urbana‐Champaign Urbana Illinois USA

**Keywords:** bubble blasting, diatom biosilica, MnO_2_ nanosheet, polymicrobial biofilm, wound dressing, wound infection

## Abstract

Treatment‐resistant wounds driven by polymicrobial biofilms are a major clinical challenge, affecting millions globally and leading to chronic inflammation, persistent pain, and poor healing outcomes. These wounds are characterized by mature biofilms reinforced by dense extracellular polymeric substances, which confer strong tolerance to conventional treatments. Despite emerging technologies, such as nanoparticles, bacteriophages, and engineered enzymes, effective clearance of established biofilms remains challenging. Here, we develop a microblasting wound dressing (µBLAST) that delivers spatially confined mechano‐chemical disruption at the tissue‐biofilm interface to remove viscoelastic biofilm matrices and promote tissue regeneration. The µBLAST is assembled by embedding MnO_2_‐doped diatom biosilica beneath an H_2_O_2_‐releasing cellulose mesh, enabling localized catalytic microbubble generation within biofilm matrices. Confined expansion and rupture of oxygen bubbles produce localized mechanical stress sufficient to dislodge mature, antibiotic‐resistant polymicrobial biofilms, while sustained H_2_O_2_ release prolongs particle activity. In a murine wound model infected with mature *P. aeruginosa* and methicillin‐resistant *S. aureus* biofilms, µBLAST treatment significantly reduces biofilm burden, accelerates re‐epithelialization, promotes hair regrowth, and mitigates inflammation. Moreover, µBLAST enhances antibiotic efficacy, suppressing biofilm regrowth even at ten‐fold reduced drug doses. These findings highlight confined mechano‐chemical biofilm disruption as a therapeutic strategy for treating mature, antibiotic‐resistant biofilm infections and promoting tissue regeneration.

## Graphical abstract

Treatment‐resistant wounds caused by polymicrobial biofilms are refractory to conventional therapies due to the dense extracellular matrices. We developed μBLAST, a microblasting wound dressing that combines MnO_2_‐doped biosilica and a H_2_O_2_‐releasing mesh to generate localized oxygen microbubbles that mechanically disrupt biofilms. In infected murine wounds, μBLAST reduced biofilm burden, enhanced antibiotic efficacy, accelerated healing, and suppressed inflammation and regrowth.

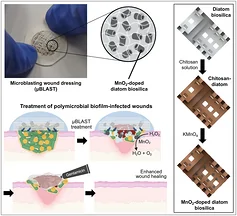

## 1 Introduction

Treatment‐resistant wounds driven by polymicrobial biofilm infection represent a major and growing clinical challenge, affecting millions of patients worldwide and imposing substantial healthcare costs. Biofilms are present in up to 80% of these non‐healing wounds [1, 2]. Such wounds contribute to chronic inflammation, persistent pain, and limited tissue regeneration. Such wounds are highly prevalent in non‐healing skin and soft‐tissue infections, particularly in aging, diabetic, and immunocompromised populations. Polymicrobial biofilm consists of communities of multiple microbial species embedded within a self‐produced, dense extracellular polymeric substances (EPS) and is implicated in the majority of chronic and recurrent infections [3, 4, 5]. The viscoelastic EPS matrix restricts the transport of antimicrobial reagents and shields embedded bacteria from host immune responses, allowing microbial cells to remain viable even under aggressive treatment [6, 7]. Within biofilms, bacterial cells often adopt a metabolic dormant state and develop adaptive resistance through polymicrobial interactions, further diminishing the efficacy of conventional antibiotics. Efforts to counteract this tolerance with frequent or repeated antibiotic treatment can inadvertently promote the emergence and spread of antibiotic‐resistant strains and stimulate virulence factor secretion [8, 9, 10, 11]. As a result, infected tissues experience prolonged inflammation, compromised tissue barrier integrity, increased discomfort and pain, and substantial delays or failure in wound healing.

Current clinical management of established biofilm‐infected wounds relies on physical or chemical removal methods, including surgical, enzymatic, autolytic, nanoparticle‐based, or hydrosurgical debridement [12, 13, 14, 15, 16]. These methods are combined with topical or systemic antimicrobial therapies. Conventional approaches, including irrigation and debridement, remain the standard of care in clinical practice because they are simple and accessible for reducing planktonic bacteria burden, flushing away wound debris and exudate, and removing necrotic tissue, thereby facilitating subsequent healing processes [17, 18]. However, these interventions often result in incomplete biofilm removal, collateral damage to surrounding healthy tissue, and significant patient discomfort. Emerging technologies such as ultrasonic devices and bubble‐generating microparticles have been explored as non‐invasive means to mechanically disrupt biofilms [19, 20, 21, 22]. While conceptually promising, such systems are limited by insufficient spatial confinement, bubble buoyancy, and reduced penetration into dense, viscoelastic biofilm matrices, restricting their effectiveness against mature polymicrobial biofilms embedded within tissue environments.

Here, we address this unmet challenge by introducing a microblasting wound dressing (µBLAST) designed to deliver spatially confined mechano‐chemical disruption at the tissue‐biofilm interface. The µBLAST is constructed in situ by embedding MnO_2_‐doped diatom biosilica (MnO_2_‐biosilica) beneath a H_2_O_2_‐releasing cellulose mesh (Movie S1). We hypothesize that this architecture enables self‐propulsion and penetration of MnO_2_‐biosilica particles, which are activated by the catalytic decomposition of H_2_O_2_, to generate oxygen bubbles within biofilm matrices. Bubble expansion within the EPS, followed by confined rupture, generates localized mechanical stress sufficient to dislodge and fragment mature biofilms, while sustained H_2_O_2_‐release prolongs particle activity over therapeutically relevant time scales. This localized microblasting behavior is conceptually analogous to directed cushion blasting used in controlled excavation, where spatial confinement amplifies mechanical efficiency while limiting collateral damage. In the biological context, this confined mechano‐chemical approach synergizes with antibiotics by enhancing drug penetration and efficacy, thereby reducing inflammation and accelerating tissue healing.

**FIGURE 1:**
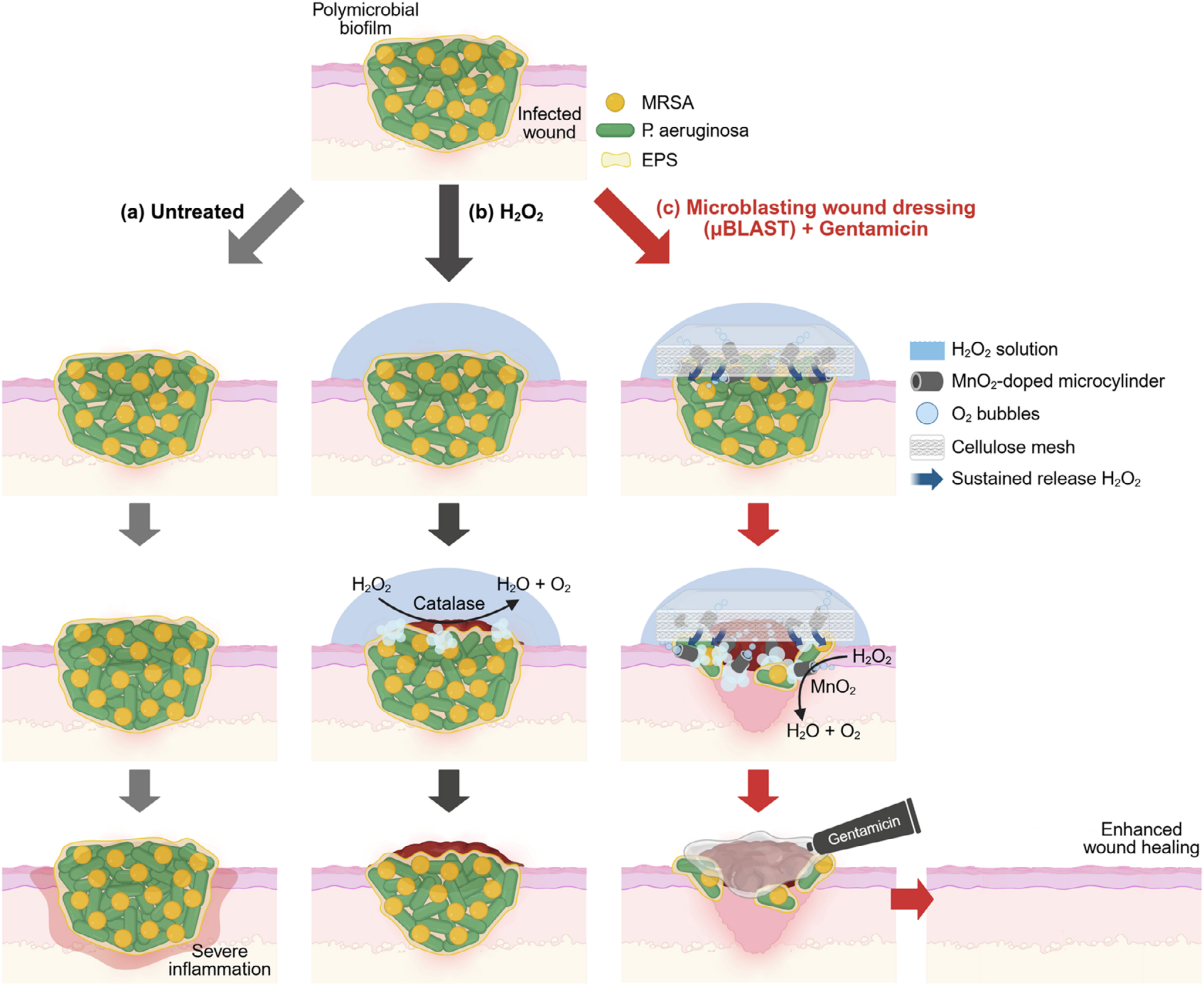
Schematic illustration of open wounds infected with polymicrobial biofilm, comparing outcomes without and with treatment using 3% H_2_O_2_ solution, and µBLAST and antibiotics. (a) In the absence of treatment, biofilm persists within the open wound, impeding closure and triggering inflammation. (b) Treatment with 3% H_2_O_2_ solution disrupts only the superficial layer of the biofilm, failing to fully eliminate the biofilm and resolve inflammation. (c) Sequential treatment with µBLAST, which consists of MnO_2_‐biosilica and cellulose mesh releasing H_2_O_2_, generates localized and sustained bubble bursts within the biofilm, enabling its effective biofilm removal. Subsequent antibiotic administration suppresses residual bacterial regrowth, facilitating re‐epithelialization and wound healing.

We examined this hypothesis using a polymicrobial biofilm model comprising *Pseudomonas aeruginosa* (PA) and methicillin‐resistant *Staphylococcus aureus* (MRSA). Mature, treatment‐resistant biofilms were generated through extended culture, resulting in increased biofilm density, yield stress, elasticity, and antibiotic resistance, as confirmed through imaging, rheological measurements, and proteomic analyses. The performance of the µBLAST was characterized by quantifying bubble dynamics, biofilm disruption, and antibiotic efficacy using acoustic measurements, optical coherence tomography (OCT), and confocal microscopy in conjunction with biofilm analysis software. Therapeutic efficacy was further evaluated in a mouse cutaneous wound model infected with mature, viscoelastic, polymicrobial biofilms for three days, with wound contraction inhibited by a splint to more closely recapitulate impaired human wound healing. Treatment outcomes were assessed by measuring biofilm regrowth, wound closure, inflammatory response, and tissue regeneration. Overall, this study establishes innovative confined mechano‐chemical biofilm disruption as a therapeutic strategy for treating mature polymicrobial wound infections and promoting tissue repair (Figure 1).

## 2 Results

### 2.1 Co‐Culture of PA and MRSA Reinforces Biofilm Metabolism, EPS, and Yield Stress

**FIGURE 2:**
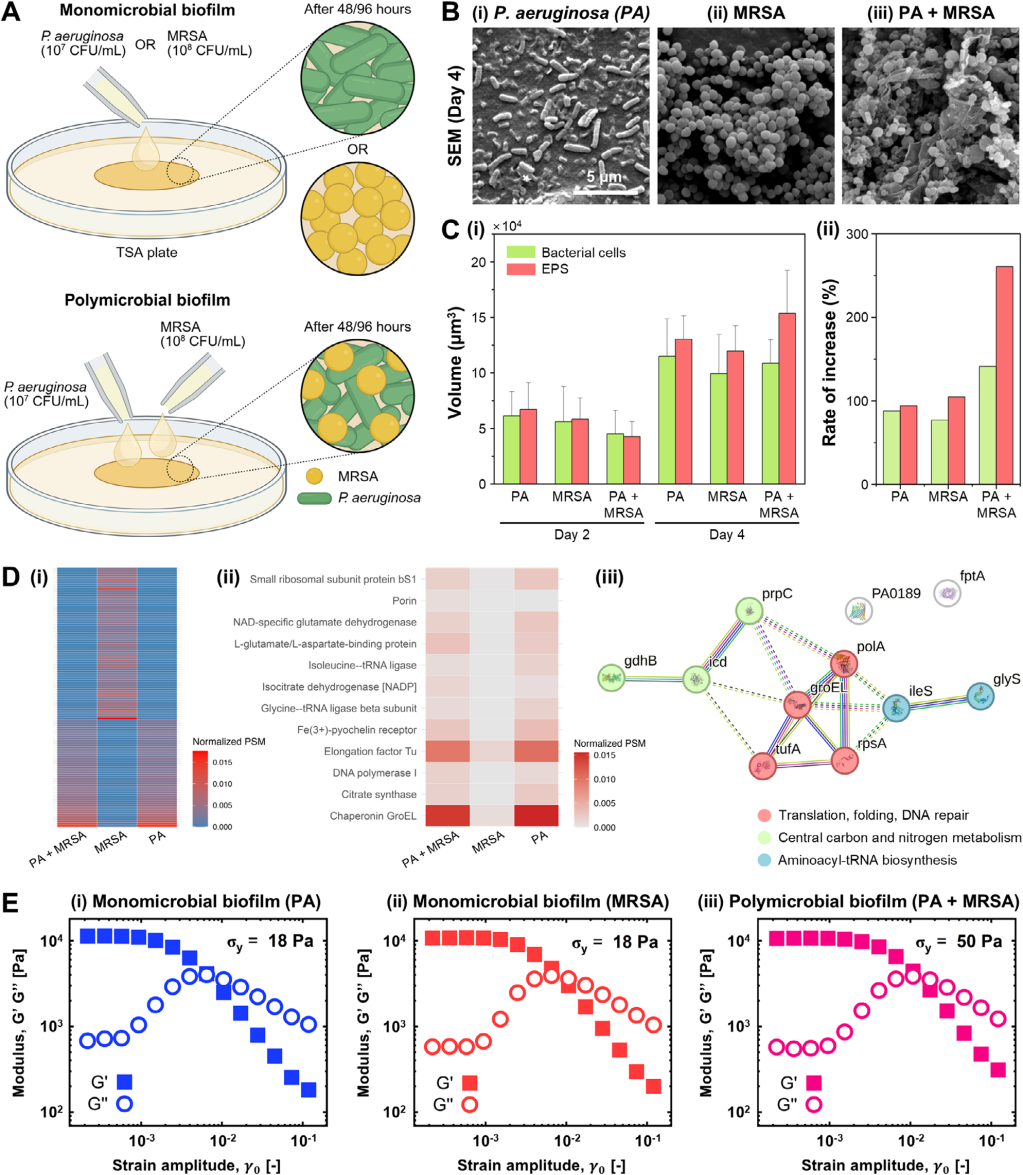
Biochemical and biomechanical characterization of monomicrobial and polymicrobial biofilms. (A) Schematic illustration of monomicrobial and polymicrobial biofilm cultures. Monomicrobial biofilms were cultured with PA or MRSA, while polymicrobial biofilms were co‐cultured with both species. (B) SEM images of biofilms formed by (i) PA, (ii) MRSA, and (iii) PA+MRSA, showing enhanced EPS accumulation in the polymicrobial biofilm. (C) (i) Quantification of biofilm biomass volume (segmented into bacterial cells and EPS) using BiofilmQ analysis of confocal z‐stacks (see Figure S5), (ii) EPS volume increase rates across conditions, showing the synergistic increase in polymicrobial biofilms. Values and error bars represent the average and standard deviation of three samples per condition. (D) Proteomic analysis of the biofilms; (i) Heatmap of normalized peptide‐spectrum match (PSM) values (> 0.0025) from label‐free quantitative proteomics comparing protein expression profiles between biofilm types, (ii) Top 15 proteins with the greatest increase in normalized PSM values in polymicrobial vs. monomicrobial PA biofilms, (iii) STRING‐based protein‐protein interaction network analysis of polymicrobial biofilm‐enriched proteins, highlighting clustering in translation, metabolism, and stress response pathways. (E) Rheological analysis of biofilms under strain sweep test. Storage modulus (G′) and loss modulus (G″), and yield stress (σ_*y*_) were measured for (i) monomicrobial PA, (ii) monomicrobial MRSA, and (iii) polymicrobial PA+MRSA biofilms. Polymicrobial biofilms exhibit higher σ_*y*_, as determined by the Kamani‐Donley‐Rogers (KDR) model.

Because polymicrobial infections are clinically more relevant and resistant to treatment than monomicrobial infections [23, 24, 25, 26], we first characterized biofilms formed by *Pseudomonas aeruginosa* (PA) and methicillin‐resistant *Staphylococcus aureus* (MRSA) co‐culture (Figure 2A). SEM and confocal microscopy revealed that polymicrobial biofilms contained a higher abundance of extracellular polymeric substances (EPS) than monomicrobial biofilms (Figure 2B and Figure S1). Quantitative analysis of confocal images from 2‐ and 4‐day cultures confirmed that polymicrobial biofilms exhibited more significant increases in bacterial cell and EPS volumes through four days of culture, compared with monomicrobial biofilms (Figure 2C).

Proteomic analysis further identified subsets of proteins that were significantly upregulated in polymicrobial biofilms compared to monomicrobial PA biofilms (Figure 2D). Since proteins expressed by each species differ in cross‐species analysis, quantitative analysis was performed using normalized peptide‐spectrum match (PSM) counts. The heatmap (Figure 2D‐i) shows that the overall protein expression patterns of the monomicrobial PA and polymicrobial PA‐MRSA biofilms are broadly similar. However, focused comparison revealed a subset of proteins with markedly elevated PSM values in the polymicrobial biofilm (Figure 2D‐ii). STRING analysis uncovered dense protein–protein interaction (PPI) networks enriched in translational and metabolic pathways (Figure 2D‐iii), consistent with enhanced EPS production. Moreover, polymicrobial biofilms exhibited upregulation of virulence‐associated proteins implicated in tissue damage and host immune modulation, underscoring a functional shift rather than broad proteomic divergence (Figure S2). Specifically, elastase (involved in extracellular matrix degradation), LptH (responsible for LPS transport to the outer membrane), HCP1 (linked to inflammatory and cytotoxic effects), and hemolysin (which promotes cell membrane lysis) were significantly increased in polymicrobial biofilms relative to monomicrobial PA biofilms.

Rheological characterization was performed using amplitude sweeps that probed the transition from the small‐amplitude linear viscoelastic regime to the large‐amplitude nonlinear regime (Figure 2E and Figure S3). The polymicrobial biofilm exhibited nonlinear behavior at larger strain amplitudes compared to monomicrobial biofilms of PA and MRSA. With these curves, the yield stress, σ_*y*_, shown in Table S1, was determined using the Kamani‐Donley‐Rogers (KDR) model, which describes yielding as a continuous transition via a rate‐dependent relaxation time [27, 28]. Based on this analysis, the polymicrobial biofilm exhibited a significantly higher σ_*y*_ than either of the monomicrobial biofilms.

### 2.2 µBLAST Produce Sustained Bubble Activity and Low‐to‐Medium‐Frequency Acoustic Emissions

**FIGURE 3:**
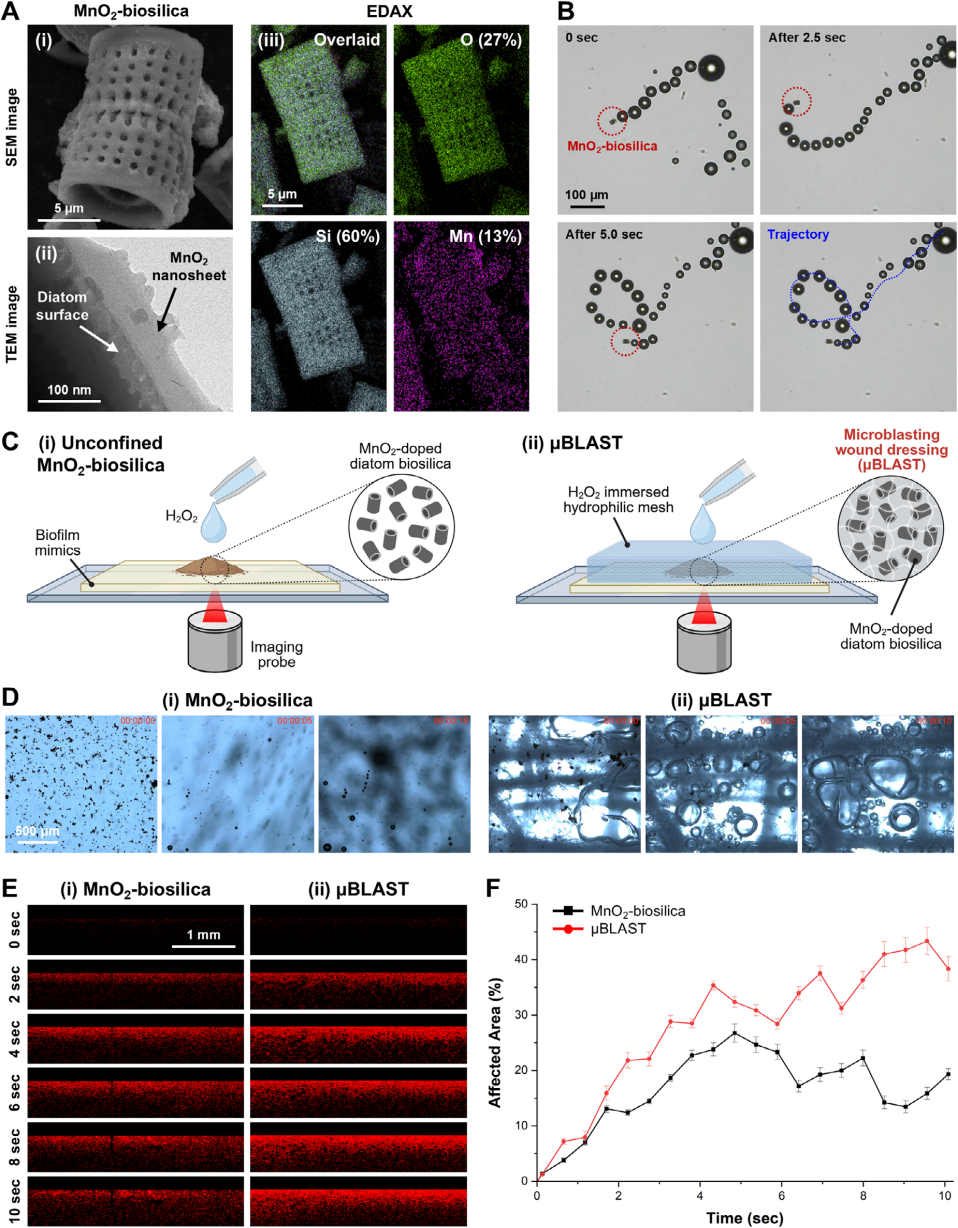
Characterization of MnO_2_‐biosilica and the µBLAST and their disturbance of biofilm mimics. (A) (i) SEM image of the MnO_2_‐biosilica in the form of a porous cylinder with a central channel, (ii) TEM image showing 1–2 nm‐thick MnO_2_ nanosheets on MnO_2_‐biosilica, (iii) EDAX maps of MnO_2_‐biosilica showing oxygen (green), silica (blue), and manganese (violet). (B) Self‐propulsion trajectories of MnO_2_‐biosilica activated in 3% H_2_O_2_ solution. (C) Schematic of the OCT imaging setup used to monitor disturbance generated by (i) unconfined MnO_2_‐biosilica and (ii) µBLAST. µBLAST comprises a cellulose fiber mesh hydrated with H_2_O_2_ and loaded with MnO_2_‐biosilica particles. (D) Bubble generation difference between (i) unconfined MnO_2_‐biosilica and (ii) µBLAST in a microscope image focused on the substrate surface. (E) Speckle‐variance OCT maps of biofilm immediately after treatment with (i) unconfined MnO_2_‐biosilica and (ii) µBLAST after activating with H_2_O_2_. The speckle variance signal was computed from sequential cross‐sectional OCT images acquired within the same region of interest. The higher intensity indicates greater disturbance. (F) Quantification of the affected area over time derived from speckle variance maps. Values and error bars represent the average and standard deviation of three samples per condition in (F).

To address the current challenge of eliminating polymicrobial biofilm, we assembled µBLAST by embedding MnO_2_‐doped diatom biosilica (MnO_2_‐biosilica) beneath a hydrophilic cellulose mesh. MnO_2_‐biosilica was prepared by immersing cylindrical, porous diatom biosilica particles coated with chitosan in KMnO_4_ solution (Figure S4). This reaction yielded porous diatoms uniformly covered with MnO_2_ nanosheets (Figure 3A‐i, ‐ii). These nanosheets comprised 13% of the total mass of MnO_2_‐biosilica, as confirmed by transmission electron microscopy (TEM) and energy‐dispersive X‐ray analysis (EDAX) combined with scanning electron microscopy (SEM, Figure 3A‐iii). MnO_2_‐biosilica was activated upon the addition of 3% H_2_O_2_ solution, initiating self‐propulsion (Figure 3B). Individual MnO_2_‐biosilica particles generated oxygen bubbles predominantly through their central hollow channel (Movie S2). To evaluate the reproducibility of MnO_2_ loading, SEM‐EDAX analysis was performed on MnO_2_‐diatom particles prepared from independently prepared batches. The results showed a comparable Mn/Si ratio across independently prepared batches, indicating reproducible MnO_2_ coating on the diatom biosilica (Figure S4C). The catalytic activity of MnO_2_‐diatoms was further assessed by measuring oxygen generation upon exposure to H_2_O_2_ (Figure S4D). Oxygen generation profiles across different batches showed consistent catalytic performance.

µBLAST were activated by hydrating cellulose fiber mesh embedded with MnO_2_‐biosilica with 3% H_2_O_2_ solution. To model the mechanical perturbation capacity of activated µBLAST, we placed them on agarose gels that mimic the viscoelastic properties of biofilms (Figure 3C). In microscopic images with a focus on the substrate surface, µBLAST localized bubble generation and rupture near the substrate, in contrast to unconfined MnO_2_‐biosilica that rapidly dispersed due to the buoyancy effect. (Figure 3D, Movies S3, and S4). Speckle variable maps were also computed from a 10‐second sequence acquired at a fixed field of optical coherence tomography view immediately after treatment with either µBLAST or unconfined MnO_2_‐biosilica (Figure 3E and Figure S5). Speckle variance quantifies the temporal intensity of perturbation at each pixel and visualizes the area of the biofilm mimics that were affected during treatment, enabling the evaluation of structural deformation over time. Relative to the baseline, µBLAST disturbed a larger and deeper region of the mimic, consistent with quantitative area analysis (Figure 3F). Specifically, µBLAST rapidly increased the affected area to 32% within 4 s and continued to expand to a maximum of 43%. In contrast, unconfined MnO_2_‐biosilica initially affected ∼22%, peaked at 28% at 5 s, and then declined over the next 5 s.

**FIGURE 4:**
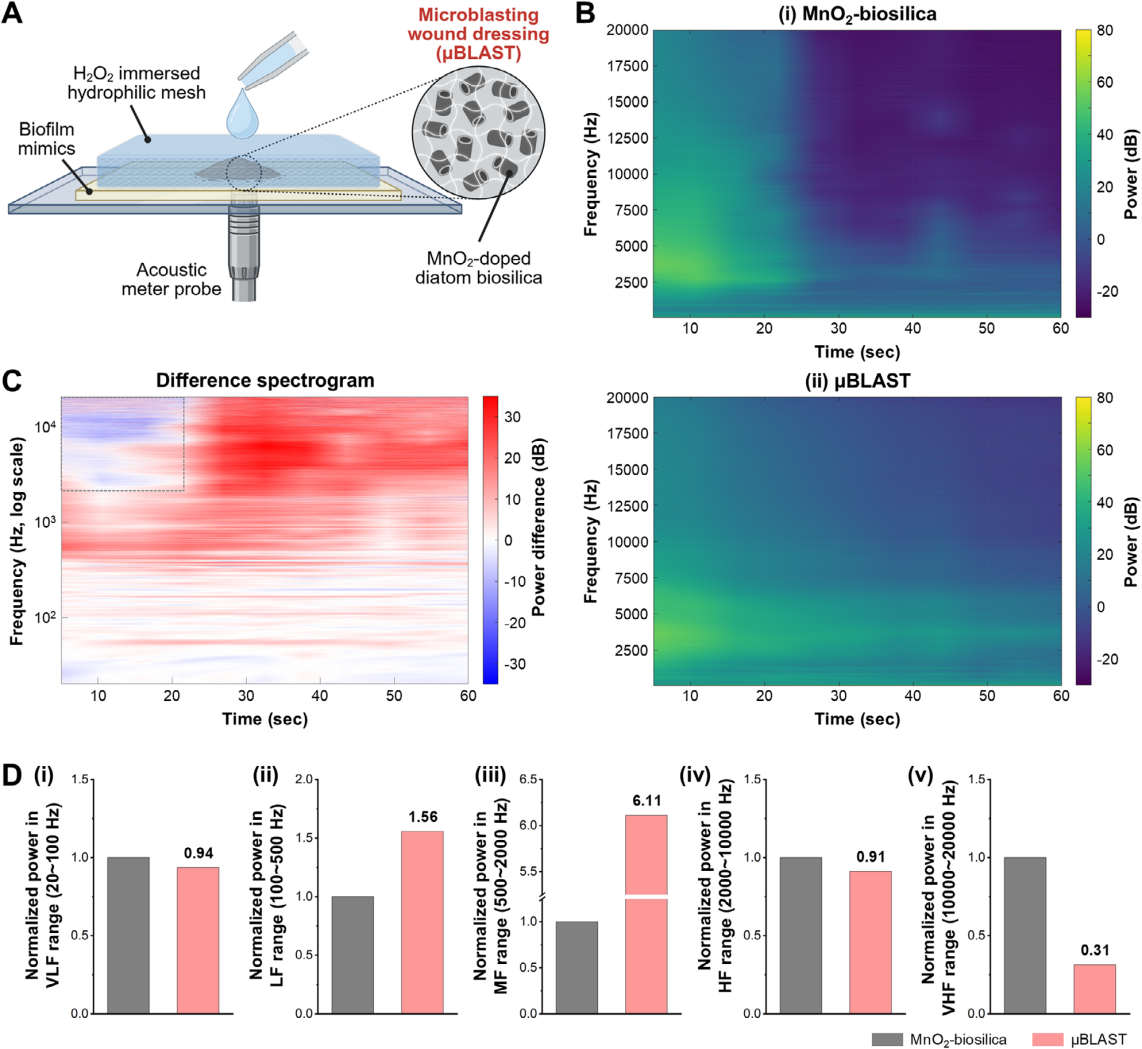
Acoustic analysis of oxygen bubble rupture dynamics generated by unconfined MnO_2_‐biosilica and the µBLAST. (A) Schematic illustration of the experimental setup used for sound recording of bubble rupture. (B) Spectrograms of bubble‐bursting sounds generated by (i) unconfined MnO_2_‐biosilica and (ii) µBLAST. Both spectrograms display the frequency profile of sounds generated by bubble rupture events at different time points. The color bar indicates the corresponding acoustic power at each frequency. (C) Difference spectrogram illustrating frequency ranges with dominant acoustic energy for each condition (Blue represents frequencies that are dominant in the unconfined MnO_2_‐biosilia, while red represents frequencies that are dominant in the µBLAST condition. (D) Comparison of normalized total acoustic power across frequency bands between the two treatments; (i) 20–100 Hz, (ii) 100–500 Hz, (iii) 500–2000 Hz, (iv) 2000–10 000 Hz, and (v) 10 000–20,000 Hz ranges. All values are normalized to the unconfined MnO_2_‐biosilica group.

The strong mechanical perturbation by µBLAST correlated with a sustained low‐frequency acoustic emission. An acoustic meter placed beneath the µBLAST recorded sounds generated by oxygen bubble rupture during H_2_O_2_ decomposition (Figure 4A). Time‐resolved spectrograms (Figure 4B) created using raw traces (Figure S6) showed that µBLAST produced sustained higher acoustic emissions in the 20–2000 Hz band than unconfined MnO_2_‐biosilica. A difference spectrogram (µBLAST vs. unconfined MnO_2_‐biosilica), derived from spectrograms shown in Figure 3B, highlights regions of relative dominance: red marks stronger acoustic emission from µBLAST, while blue marks dominance of the unconfined MnO_2_‐biosilica (Figure 4C). Above 2000 Hz, the unconfined MnO_2_‐biosilica briefly led (∼ 0–15 s), but after 25 s, µBLAST surpassed it across all frequencies and persisted for 2 min, consistent with faster H_2_O_2_ consumption by the unconfined MnO_2_‐biosilica (Figure S7). With a single application of H_2_O_2_, acoustic output from unconfined MnO_2_‐biosilica declined rapidly, whereas µBLAST maintained sound generation. Under repeated H_2_O_2_ administration every 30 s, µBLAST produced continuous sound while the unconfined MnO_2_‐biosilica showed distinct initiation and termination of sound.

Quantitative analysis of power spectra confirmed that µBLAST produce sustained low‐frequency acoustic emissions compared with unconfined MnO_2_‐biosilica. To evaluate this, the frequency spectrum was divided into five standard acoustic bands: very low frequency (VLF), low frequency (LF), medium frequency (MF), high frequency (HF), and very high frequency (VHF). The total acoustic power generated during H_2_O_2_ decomposition was measured for each band (Figure 4D). These bands correspond to bubble rupture dynamics driven by the decomposition of H_2_O_2_ by either µBLAST or unconfined MnO_2_‐biosilica. Acoustic power in the VLF and HF bands was comparable between the two treatment conditions. However, µBLAST generated significantly greater power in the LF and MF ranges. In contrast, unconfined MnO_2_‐biosilica exhibited greater power in the VHF band. These findings indicate substantial differences in the bubble size distributions produced by the two systems, with the µBLAST promoting the formation and collapse of larger bubbles.

### 2.3 µBLAST Disrupts EPS Structure and Leads to the Eradication of In Vitro Polymicrobial Biofilm, Combined With Antibiotics

**FIGURE 5:**
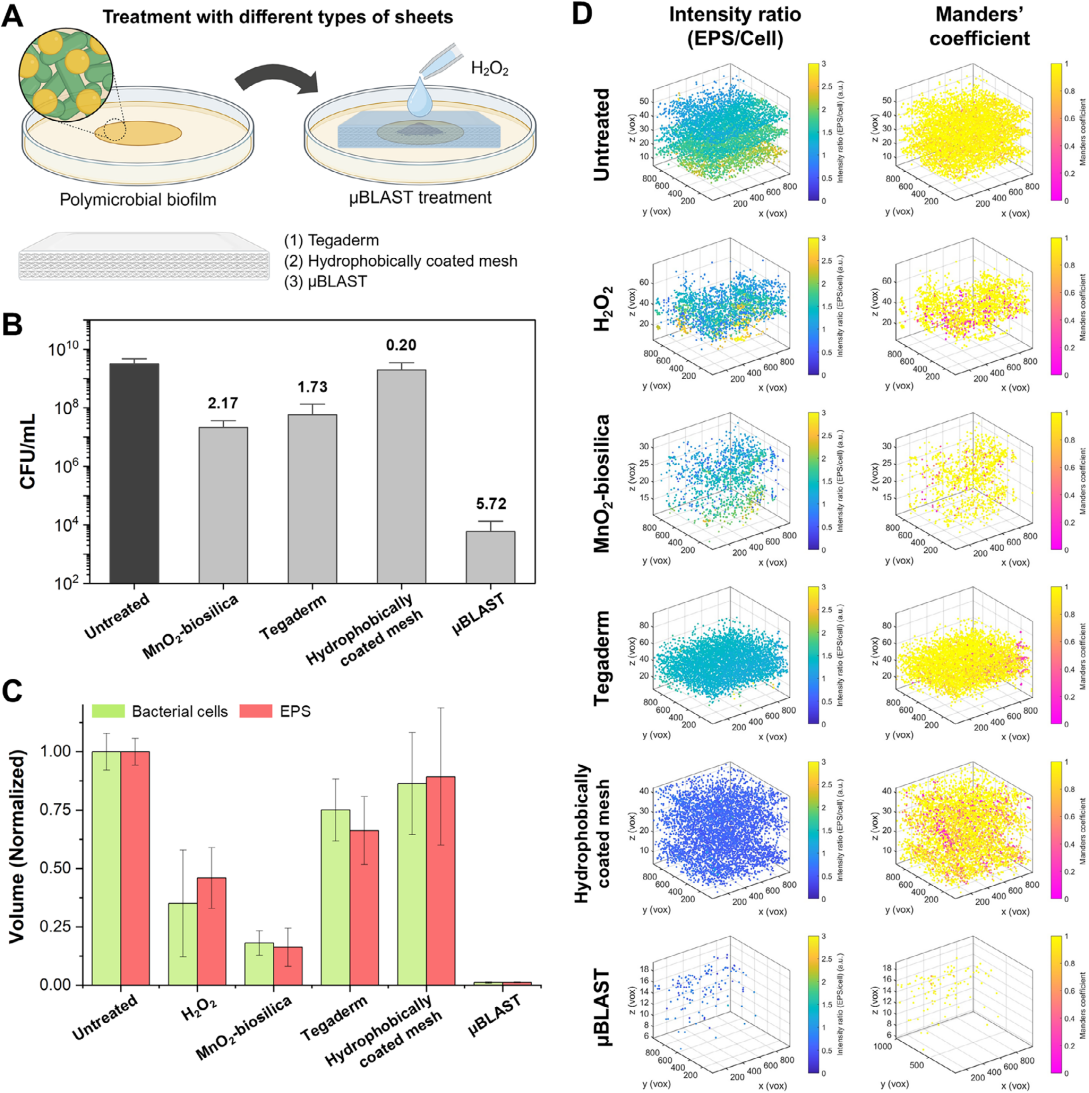
Evaluation of PA‐MRSA polymicrobial biofilm removal in vitro by µBLAST. (A) Schematic of the in vitro experimental setup for assessing µBLAST efficacy against PA‐MRSA polymicrobial biofilms. Biofilms subjected to different treatments were analyzed by quantifying residual biofilm volume and enumerating CFU of viable cells. Treatment groups include: unconfined MnO_2_‐biosilica (denoted MnO_2_‐biosilica), MnO_2_‐biosilica covered with Tegaderm and overlaid with 3% H_2_O_2_ solution (denoted Tegaderm), MnO_2_‐biosilica loaded in hydrophobically coated cellulose mesh overlaid with 3% H_2_O_2_ solution (denoted hydrophobic‐coated mesh), and µBLAST. (B) Quantification of bacterial load after each treatment condition. Numbers within the plot indicate log reduction relative to the untreated control. (C) Normalized biomass volumes of bacterial cells and EPS following each treatment, quantified from immunostained confocal images (see Figure S9). (D) 3D plots of EPS‐to‐cell intensity ratio and the Manders’ coefficients reconstructed from confocal images using Biofilm‐Q software after different treatments. Values and error bars represent the average and standard deviation of six samples per condition in (B), and three samples per condition in (C).

The localized and sustained oxygen bubble generation and rupture by the µBLAST for 2 min served to reduce the number of viable bacterial cells in polymicrobial biofilms. For this study, we formed polymicrobial PA‐MRSA biofilms on agar gel in vitro for 3 days. The resulting biofilms were treated with several conditions to examine the combined effects of confinement for MnO_2_‐biosilica and the release profile of H_2_O_2_ from the dressing (e.g., polyurethane film, hydrophobically modified cellulose mesh, and hydrophilic cellulose mesh), each of which was loaded with MnO_2_‐biosilica (Figure 5A). Quantification of CFUs showed that unconfined MnO_2_‐biosilica achieved a 2.2‐log reduction in bacterial load, while MnO_2_‐biosilica loaded with polyurethane film with nano‐sized pores or the hydrophobic‐coated cellulose mesh resulted in 1.7‐log reduction and 0.2‐log reduction, respectively, relative to the untreated control (Figure 5B). Notably, the µBLAST composed of MnO_2_‐biosilica and hydrophilic cellulose mesh produced a significant 5.7‐log reduction. We also performed control experiments to decouple the effects of MnO_2_, oxygen bubble generation, and µBLAST‐mediated mechanical debridement (Figure S8A). Treatments with diatom biosilica, MnO_2_ powder, and MnO_2_‐biosilica alone did not result in significant reductions in bacterial viability, indicating negligible intrinsic antibacterial effects of the materials. Exposure to 3% H_2_O_2_ alone resulted in a 2.96‐log reduction in CFU, while catalase‐biosilica combined with H_2_O_2_, which generates oxygen on the biofilm surface through enzymatic decomposition, led to a 2.37‐log reduction. Although these treatments achieved > 99% bacterial reduction, µBLAST achieved an additional 1.9–2.5 log reduction in CFU, corresponding to substantially greater biofilm removal efficacy. These results demonstrate that oxygen bubble generation alone is insufficient to explain the enhanced antibacterial efficacy of µBLAST. Instead, the improved performance arises from the localized generation and dynamic evolution of oxygen bubbles by MnO_2_‐doped biosilica that enter the 3D biofilm matrix through self‐propulsion. Such dynamic bubble evolution within the 3D biofilm matrix can mechanically disrupt the biofilm structure while simultaneously enhancing H_2_O_2_ transport into the 3D biofilm. To further evaluate strain‐specific antibacterial effects, additional experiments were performed using monomicrobial biofilms of *P. aeruginosa* and MRSA under identical treatment conditions (Figure S8B). In monomicrobial biofilms, µBLAST treatment resulted in significant reductions in bacterial burden for both *P. aeruginosa* and MRSA, consistent with the overall trends observed in polymicrobial biofilms.

µBLAST was also most effective in removing both cells and EPS of biofilms, as shown by confocal images stained for proteins on the cell membrane and polysaccharides in EPS (Figure S9). Quantification of cells and EPS volumes showed the most significant decreases in both values after treating biofilms with µBLAST (Figure 5C). Such a degree of reduction was more statistically significant compared to the biovolume of biofilms treated with unconfined MnO_2_‐biosilica. We further analyzed cell‐matrix interactions using intensity ratio mapping and Manders’ coefficient distribution in 3D biofilms (Figure 5D). These two quantified values, obtained using Biofilm‐Q analysis software [29], represent the relative abundance of matrix‐to‐cell signal and the fraction of cells that spatially overlap with the matrix, respectively. In this regard, the untreated biofilm showed that the matrix becomes denser along the depth, as indicated by the higher intensity ratio value towards the bottom. In contrast, the cells and matrix are tightly associated, as noted by the Manders’ coefficient, which approaches 1.0. 3D plots of biofilms post‐treatment with µBLAST disclosed the decreased intensity ratio of the residual parts compared to untreated biofilms. Unconfined MnO_2_‐biosilica also decreased the intensity ratio moderately, but the intensity ratio of residual biofilm on the bottom side remained similar to the untreated biofilm. Using polyurethane film or hydrophobically modified cellulose mesh as the part releasing H_2_O_2_ resulted in minor decreases in the matrix and cell number compared to the untreated biofilm, although the intensity ratio became lower than that of the untreated biofilm.

**FIGURE 6:**
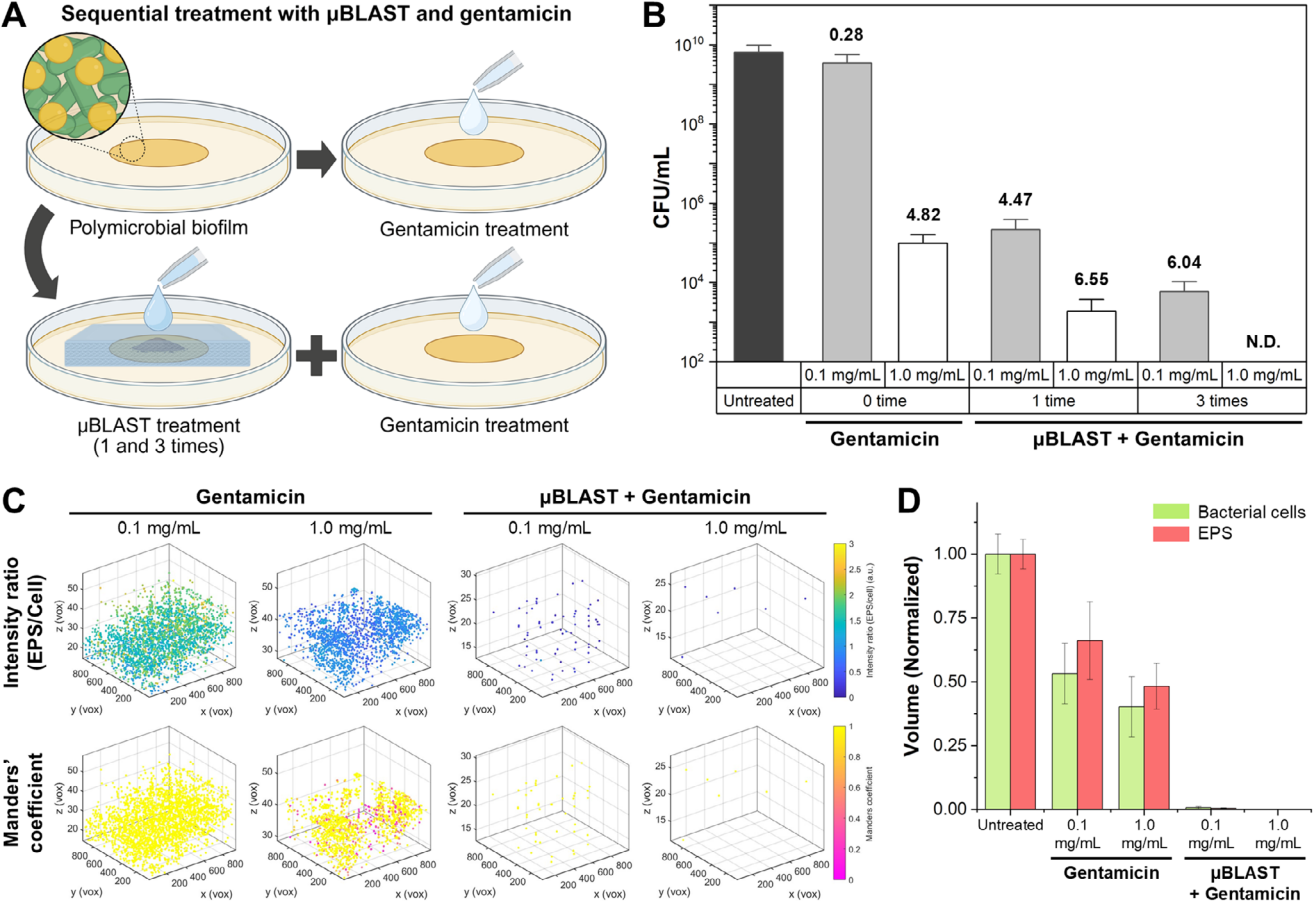
Evaluation of polymicrobial biofilm removal in vitro by the combination of µBLAST pretreatment and antibiotics (gentamicin). (A) Schematic of the in vitro experimental setup for assessing sequential treatment efficacy of µBLAST and gentamicin against polymicrobial PA‐MRSA biofilms. Biofilms subjected to different treatments were analyzed by quantifying residual biofilm volume and enumerating CFU of viable cells. (B) Quantification of bacterial load after each treatment condition varied with µBLAST pretreatment and gentamicin doses. Numbers within the plot indicate log reduction relative to the untreated control. N.D. represents non‐detectable. (C) 3D plots of EPS‐to‐cell intensity ratio and the Manders’ coefficients reconstructed from confocal images using Biofilm‐Q software after different treatments. (D) Normalized biomass volumes of bacterial cells and EPS following each treatment, quantified from immunostained confocal images (see Figure S11). Values and error bars represent the average and standard deviation of six samples per condition in (B), and three samples per condition in (D).

Based on the observation that µBLAST can effectively disrupt and remove biofilm architecture, we further evaluated the synergistic potential of combining µBLAST with gentamicin. The combination treatment was significantly more effective in eliminating polymicrobial biofilms than gentamicin monotherapy. As shown in Figure 6A, two groups were prepared: one treated with gentamicin monotherapy, and the other pretreated with the µBLAST followed by gentamicin. The µBLAST was applied zero times (gentamicin monotherapy), once, or three times, followed by gentamicin treatment at either 0.1 mg/mL or 1.0 mg/mL. According to CFU measurements, gentamicin monotherapy at 0.1 mg/mL was ineffective in reducing the biofilm burden, whereas 1.0 mg/mL achieved a 4.8‐log reduction (Figure 6B). In contrast, when biofilms were pretreated once with the µBLAST prior to gentamicin application, a 4.5‐log reduction was achieved even at 0.1 mg/mL, and a 6.6‐log reduction was observed at 1.0 mg/mL. Furthermore, when biofilms were pretreated three times with the µBLAST, subsequent treatment with 0.1 mg/mL gentamicin resulted in a 6.0‐log reduction, while treatment with 1.0 mg/mL gentamicin reduced the bacterial load to undetectable levels. To distinguish the contribution of H_2_O_2_ from µBLAST‐mediated mechanical disruption, we compared antibacterial efficacy after H_2_O_2_ or µBLAST pretreatment prior to gentamicin treatment (Figure S10A). Treatment with H_2_O_2_ combined with gentamicin resulted in 3.79‐ and 4.92‐log reductions at 0.1 and 1.0 mg/mL, respectively. In contrast, µBLAST combined with gentamicin achieved 4.30‐log reduction at 0.1 mg/mL and complete eradication at 1.0 mg/mL. To further evaluate strain‐specific antibacterial efficacy, monomicrobial biofilms of *P. aeruginosa* and MRSA were treated with gentamicin monotherapy or in combination with µBLAST (Figure S10B). In *P. aeruginosa* monomicrobial biofilms, gentamicin monotherapy resulted in a 4.28‐log reduction at 0.1 mg/mL and complete bacterial elimination at 1.0 mg/mL. µBLAST combined with gentamicin further enhanced antibacterial efficacy, achieving a 7.00‐log reduction at 0.1 mg/mL and complete elimination at 1.0 mg/mL. Similarly, in MRSA monomicrobial biofilms, gentamicin monotherapy resulted in 3.64‐log reduction at 0.1 mg/mL and 4.57‐log reduction at 1.0 mg/mL. In contrast, µBLAST combined with gentamicin improved bacterial reduction to 4.68 logs at 0.1 mg/mL and achieved complete elimination at 1.0 mg/mL. The three‐dimensional spatial distributions of bacterial cells and EPS reconstructed from confocal microscopy images (Figure 6C and Figure S11) and the corresponding biovolume quantification (Figure 6D) also confirm that the combination treatment resulted in more effective biofilm removal than gentamicin monotherapy.

We also performed RT‐qPCR analysis to evaluate the relative mRNA expression levels of biofilm‐ and stress‐associated genes in *P. aeruginosa* and MRSA (Figure S12). RT‐qPCR analysis revealed that µBLAST pretreatment followed by gentamicin significantly downregulated *pelA*, *lasI*, and *rhlI* expression in *P. aeruginosa* compared with gentamicin alone, indicating disruption of EPS production and quorum‐sensing pathways associated with biofilm maintenance. In MRSA, sequential treatment with µBLAST and gentamicin decreased expression of *fnbA* compared with gentamicin alone, indicating reduced adhesion capacity. In contrast, sequential treatment upregulated *icaA* and *vraR*, suggesting activation of stress‐responsive and biofilm‐reformation pathways. These results suggest that µBLAST facilitates antibiotic action by disrupting biofilm structure and exposing embedded bacteria, thereby inducing stress‐associated transcriptional responses.

### 2.4 µBLAST Removes Polymicrobial Biofilm in the In Vivo Wound Infection Model and Enhances Wound Healing

**FIGURE 7:**
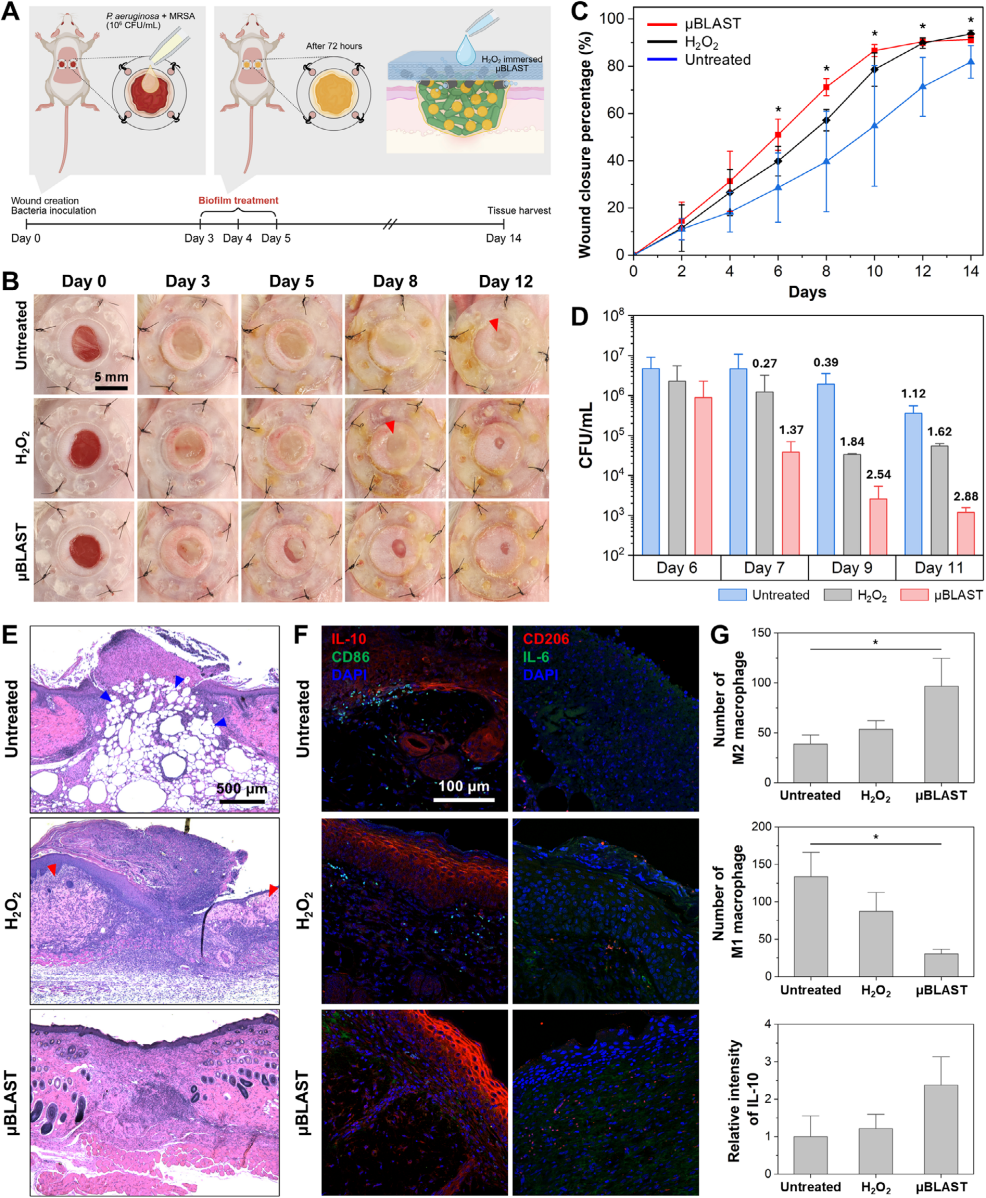
Evaluation of the in vivo efficacy of the µBLAST for removing polymicrobial biofilms in a wound infection model and promoting skin regeneration. (A) Schematic representation of the treatment timeline and in vivo evaluation of the µBLAST in a mouse wound infection model. (B) Representative photographs of wounds from each treatment group (untreated, 3% H_2_O_2_ solution, and µBLAST) on Days 4, 8, and 12. Red arrows indicate areas of persistent biofilm on the wound surface. (C) Quantitative analysis of wound closure over 14 days. * indicates the significant difference in values relative to the µBLAST condition with the untreated condition (*p* < 0.05). (D) Quantification of bacterial load in infected wounds, presented as CFU counts. Numbers indicate the log reduction value compared to Day 6 within each group. (E) Representative H&E‐stained images of wounded tissue harvested on Day 14. Blue arrows in the untreated group represent adipose tissue accumulation, while red arrows in the 3% H_2_O_2_‐treated group indicate neutrophil infiltration. (F) Immunofluorescence images of wounded tissue stained for pro‐inflammatory (CD86, IL‐6) and anti‐inflammatory (CD206, IL‐10) markers, captured on Day 14. (G) Quantitative fluorescence intensity analysis of IL‐10 from the images shown in (F), F4/80^+^CD86^+^ M1 macrophages and F4/80^+^CD206^+^ M2 macrophages from the images shown in Figure S14. * indicates the statistically significant difference between treatment groups (*p* < 0.05). Values and error bars represent the average and standard deviation of four samples per condition in (C), and three samples per condition in (D) and (G). Each sample was harvested from an independent animal.

The µBLAST demonstrated effective removal of thick, solid polymicrobial biofilms in cutaneous wounds, thereby promoting early wound closure and enhancing tissue regeneration. To establish a thick, virulent, and mechanically robust polymicrobial biofilm infection, PA and MRSA were simultaneously inoculated into full‐thickness wounds and allowed to mature for three days, as performed in vitro (Figure 7A). Wound contraction was restricted using splints. By Day 3, the open wounds were filled with yellow‐colored biofilms that resisted mechanical disruption from mouse movement (Figure 7B). Wounds were then left untreated, treated with 3% H_2_O_2_ solution, or treated with the µBLAST for three consecutive days. This repeated treatment aimed to suppress biofilm regrowth, a critical factor that delays wound healing. Throughout the 14‐day observation period, untreated wounds retained biofilms persistently. The conventional 3% H_2_O_2_ treatment had a minimal impact, with intact biofilms remaining until Day 8, allowing delayed wound closure. Between Days 8 and 12, some physical detachment of the biofilm occurred. In contrast, µBLAST treatment resulted in significant biofilm clearance as early as Day 4 after the initial application. Continued treatment effectively inhibited biofilm regrowth and accelerated wound closure, as confirmed quantitatively (Figure 7C).

Consistent with visual observation, the µBLAST also significantly reduced bacterial load in biofilms. CFU analysis of wound exudates was performed following treatment (Figure 7D). The values displayed above each bar indicate the log reduction in bacterial load compared to CFU levels on Day 6 for each treatment group. Neither the untreated group nor the H_2_O_2_‐treated group showed a significant reduction in CFUs. In contrast, wounds treated with the µBLAST exhibited a progressive reduction in CFUs, culminating in a significant 2.9‐log reduction by Day 11.

Histological analysis on Day 14 further demonstrated enhanced skin regeneration in the µBLAST‐treated group. Hematoxylin and eosin (H&E) staining showed that untreated wounds were predominantly closed by excessive adipose tissue (Figure 7E), while H_2_O_2_‐treated wounds exhibited hyperkeratosis and abundant granulation tissue. In contrast, the µBLAST‐treated wounds transformed to well‐integrated epidermal and dermal layers, with newly formed hair follicles evident within the repaired tissue.

The superior tissue regeneration correlated with a shift toward an anti‐inflammatory wound environment. Immunofluorescence staining (Figure 7F and Figures S13, and S14) showed an increased population of F4/80^+^CD206^+^ M2 macrophages and elevated IL‐10 expression in the µBLAST‐treated wounds. In contrast, wounds in the untreated and H_2_O_2_‐treated groups were dominated by F4/80^+^CD86^+^ M1 macrophage population and IL‐6 expression. Quantitative analysis further confirmed that µBLAST treatment increased M2 macrophages and IL‐10 while reducing M1 macrophage levels (Figure 7G).

### 2.5 Combination Treatment of µBLAST and Antibiotics Facilitates Synergistic Effects on Wound Healing and Biofilm Control

**FIGURE 8:**
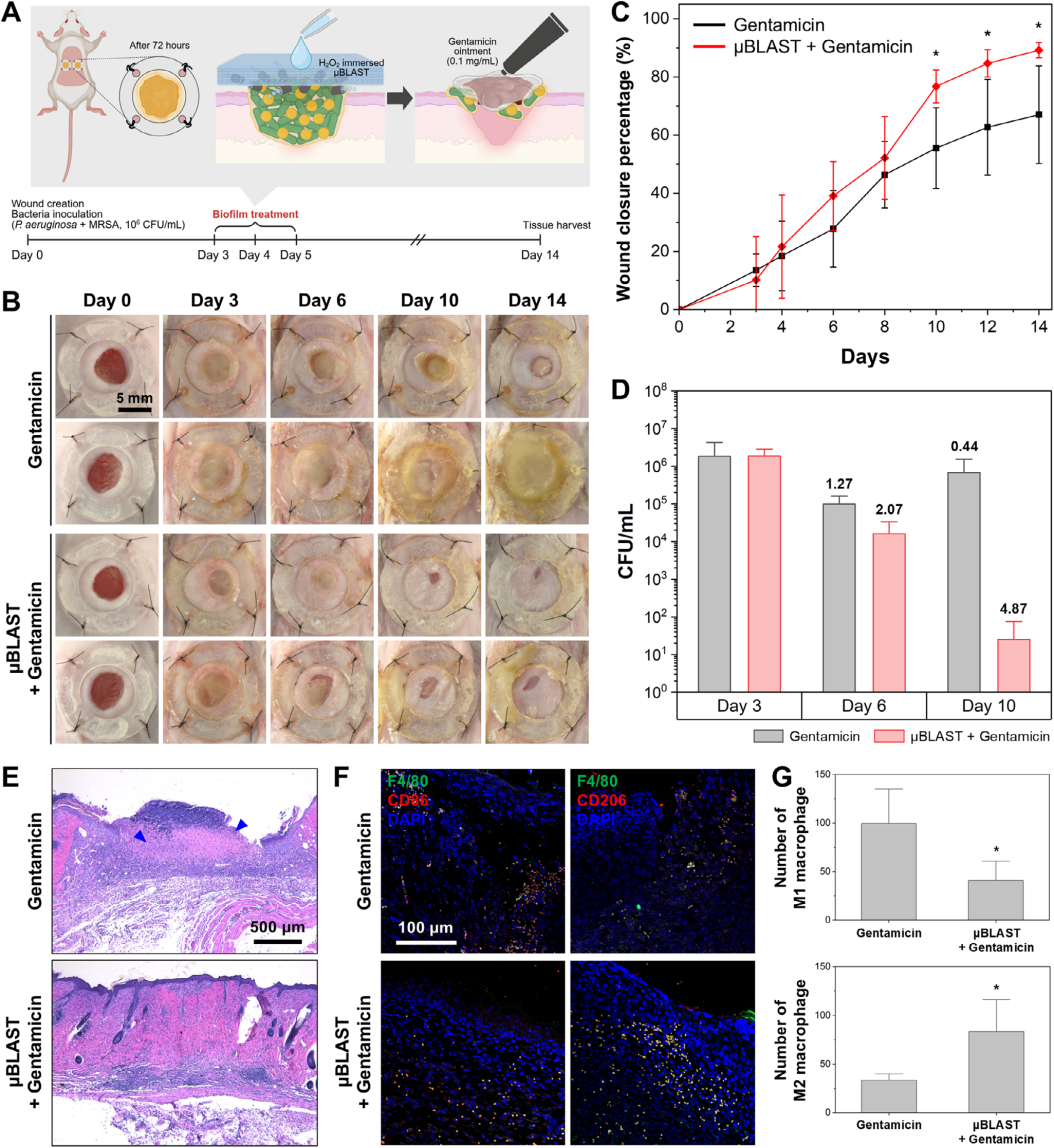
Evaluation of the synergetic effect of the µBLAST and antibiotics (gentamicin) for removing polymicrobial biofilms in a wound infection model and promoting skin regeneration. (A) Schematic representation of the treatment timeline and in vivo evaluation of the synergetic effect of the µBLAST combined with gentamicin in a mouse wound infection model. (B) Representative photographs of wounds from each treatment group (gentamicin monotherapy and the combination of µBLAST and gentamicin) on Days 3, 6, 10, and 14. Images in each row within the same treatment group represent wounds created at different skin sites. (C) Quantitative analysis of wound closure over 14 days. * indicates the significant difference between two treatment conditions (*p* < 0.05). (D) Quantification of bacterial load in infected wounds with the use of gentamicin, presented as CFU counts. Numbers indicate the log reduction value compared to Day 3 within each group. (E) Representative H&E‐stained images of wounded tissue harvested on Day 14. Blue arrows in the gentamicin monotherapy group indicate neutrophil infiltration. (F) Immunofluorescence images of wounded tissue stained for pro‐inflammatory (F4/80^+^CD86^+^) and anti‐inflammatory (F4/80^+^CD**20**6^+^) markers, captured on Day 14. (G) Quantitative analysis of F4/80^+^CD86^+^ M1 macrophages and F4/80^+^CD206^+^ M2 macrophages from the images shown in (F). * indicates the statistically significant difference between treatment conditions (*p* < 0.05). Values and error bars represent the average and standard deviation of four samples per condition in (C) and (D), and three samples per condition in (G). Each sample was harvested from an independent animal.

We next evaluated whether sequential treatment with the µBLAST and gentamicin would enhance biofilm removal and restrict regrowth more effectively than µBLAST or gentamicin alone. Following the previously established protocols, biofilm‐infected wounds were treated starting on Day 3 for three consecutive days, with wound progression monitored over 14 days (Figure 8A). Gentamicin monotherapy did not eliminate the biofilm, allowing progressive accumulation and maintenance of the infected wound microenvironment (Figure 8B). In contrast, sequential treatment with the µBLAST followed by gentamicin effectively removed biofilm, restricted regrowth, and significantly improved wound closure. By Day 10, the wound closure in the sequential treatment group was markedly greater than in the group treated with gentamicin only (Figure 8C). This synergy was also reflected in bacterial load in wounds. CFU analysis of wound exudates showed that gentamicin alone caused a modest bacterial reduction; regrowth occurred by Day 10 (Figure 8D). In contrast, the sequential treatment resulted in a robust antibacterial effect, achieving a 4.9‐log reduction in CFUs by Day 10.

Histological analysis also revealed that gentamicin monotherapy resulted in incomplete re‐epithelialization, extensive neutrophilic infiltration, and abundant granulation tissue (Figure 8E). In contrast, the sequential treatment produced well‐organized epidermal and dermal layers with hair follicles and minimal immune infiltration. Immunofluorescence imaging further revealed a predominance of F4/80^+^CD86^+^ M1 macrophages in wounds treated with gentamicin only, whereas wounds receiving the combined treatment promoted a transition to F4/80^+^CD206^+^ M2 macrophages (Figure 8F), corroborated by quantitative analysis of macrophage subtypes (Figure 8G).

## 3 Discussion

This study demonstrates µBLAST, constructed by embedding MnO_2_‐biosilica in H_2_O_2_‐releasing cellulose mesh, serves as an effective biomedical tool for removing resilient, pro‐inflammatory polymicrobial PA‐MRSA biofilms and promoting skin regeneration in infected wounds. By sustaining the activation of self‐propelling MnO_2_‐biosilica, µBLAST generates oxygen bubbles whose lower‐frequency ruptures exert a vigorous mechanical perturbation on biofilm‐simulating gels. Compared with stand‐alone self‐propelling MnO_2_‐biosilica, µBLAST more effectively removed both cells and EPS of in vitro biofilms. When applied to polymicrobial biofilm‐infected skin wounds, µBLAST eliminated most biofilm, accelerated re‐epithelialization with hair regrowth, and markedly reduced inflammatory signaling. Moreover, µBLAST improved the efficacy of antibiotics, suppressing biofilm regrowth in wounds and further enhancing tissue regeneration. Therefore, in contrast to conventional wound debridement strategies, such as surgical excision, enzymatic solutions or gels, or negative pressure wound therapy, µBLAST provides a minimally invasive, repeatable approach that integrates mechanical biofilm removal with enhanced antibacterial activity, highlighting their translational potential.

Biofilms, specifically polymicrobial biofilms, represent a major clinical challenge because of their dense EPS matrices, complex metabolic activity, and elevated mechanical rigidity compared to monomicrobial biofilms [30, 31, 32]. In our own analysis with PA and MRSA co‐cultures, we confirmed that the polymicrobial biofilms exhibited more rapid and active EPS accumulation than monomicrobial counterparts (Figure 2B,C, and Figure S1). Proteomic enrichment of proteins associated with (i) translation, folding, and DNA repair, (ii) central carbon and nitrogen metabolism, and (iii) aminoacyl‐tRNA biosynthesis biofilm suggests enhanced metabolic activity and stress adaptation, which likely contributes to their greater structural and chemical resistance (Figure 2D). Rheological analysis further revealed higher yield stress in polymicrobial biofilms, highlighting the interplay between biochemical composition and mechanical rigidity (Figure 2E and Table S1). Together, these findings suggest that as polymicrobial biofilms mature, they become increasingly resistant to both chemical and physical treatments.

This heightened resistance underscores the need for approaches that integrate biochemical reactivity with sustained mechanical disruption. To address this challenge, we developed µBLAST, designed to improve the efficacy of removing biofilm from substrates and wounded tissues. MnO_2_‐biosilica particles in µBLAST decompose hydrogen peroxide (H_2_O_2_) to generate oxygen bubbles, creating propulsive forces that drive their motion [33, 34]. Their porous cylindrical diatom structure is advantageous for self‐locomotive motion compared with spherical particles. To investigate the penetration of MnO_2_‐biosilica into biofilms, OCT imaging was performed during treatment with H_2_O_2_ alone or with MnO_2_‐biosilica particles activated by H_2_O_2_. Immediately after reaction initiation, oxygen bubbles were observed forming within the biofilm interior for the MnO_2_‐biosilica‐treated condition, while comparable bubble formation was not observed in biofilms treated with H_2_O_2_ alone (Figure S4E and Movies S5,S6). These findings indicate that MnO_2_‐biosilica particles can enter the biofilm matrix and generate bubbles locally, supporting a mechanism involving particle penetration. The generated kinetic energy enables MnO_2_‐biosilica to penetrate biofilms, while continuously producing O_2_ bubbles that expand and collapse, mechanically disrupting and detaching biofilm. However, unless confined, buoyancy caused by O_2_ bubbles restricts these particles from effectively reaching biofilms located on the bottom surface.

Loading MnO_2_‐biosilica beneath a cellulose mesh, which simultaneously releases H_2_O_2_, not only mitigated the buoyancy limitation of unconfined particles but also enhanced the biofilm removal. The mesh confined particles near the substrate, prevented flotation, and prolonged bubble generation (45 s vs. ∼ 20 s for unconfined particles; Figure S7). Rehydrating with fresh H_2_O_2_ reinitiated bubble formation. Consequently, µBLAST produced significantly greater perturbation in biofilm‐mimicking gels than unconfined MnO_2_‐biosilica, as characterized with speckle variance analysis with OCT (Figure 3E). These perturbations extended deeper and wider into the gel matrix (Figure 3F).

Acoustic analysis further disclosed that µBLAST generated oxygen bubbles collapsing at a lower frequency range (100–2000 Hz) than unconfined MnO_2_‐biosilica (Figure 4D). Because bubble collapse frequency is inversely related to bubble size according to Minnaert's equation [35, 36], these findings indicated that cellulose mesh promoted bubble coalescence and rupture at larger diameters. We propose that the hydrophilic, porous cellulose mesh absorbs water and temporarily traps bubbles within its pore spaces, facilitating their fusion. The resulting larger bubbles collapse with greater energy than those from unconfined MnO_2_‐biosilica, producing stronger low‐frequency mechanical perturbations collapse [37, 38, 39]. µBLAST sustained acoustic activity for more than 120 s, reflecting prolonged bubble generation and rupture. Together, these findings suggest that the cellulose mesh enhances mechanical energy delivery by localizing bubble collapse at the biofilm interface while also sustaining H_2_O_2_ release to maintain bubble activity. In contrast, hydrophobically coated cellulose meshes and Tegaderm limited bubble generation due to insufficient H_2_O_2_ absorption.

Such localized and sustained low‐frequency mechanical energy transfer could effectively disrupt mechanically robust polymicrobial biofilms. In vitro, µBLAST treatment reduced bacterial burden by 5.7‐log by dispersing most bacterial cells and EPS from their original sites (Figure 5B–D). Sequential treatment with µBLAST followed by gentamicin further improved clearance, outperforming either µBLAST or monotherapy. These results indicate that MnO_2_‐biosilica activated beneath the cellulose mesh penetrates biofilms and continues generating oxygen bubbles. Within the mesh pores, bubbles fuse and collapse at larger diameters, producing low‐frequency mechanical waves likely exceeding the yield stress of the polymicrobial biofilm. Continued bubble activity progressively eroded the thick biofilm, leaving residual bacterial cells more susceptible to gentamicin, which binds to the 30S ribosomal subunit, causes mistranslation and production of toxic proteins, and ultimately induces cell death [40]. Importantly, sequential treatment allowed antibiotic sparing, as evidenced by comparable log‐reduction between µBLAST followed by 0.1 mg/mL gentamicin and 1.0 mg/mL gentamicin monotherapy (Figure 6B).

The potent biofilm‐removing activity of µBLAST was also observed in biofilm‐infected wounds. To model clinically challenging infections, we established polymicrobial biofilms in puncture wounds and allowed them to mature untreated for three days, in contrast to most studies that intervene after only one day of bacterial inoculation. Proteomics analysis of in vitro polymicrobial biofilms demonstrated enrichment of virulence‐associated proteins, including elastase, LptH, HCP1, and hemolysin, all of which are known to exacerbate inflammation and impair tissue regeneration in infected wounds (Figure S2) [41, 42, 43, 44].

Interestingly, µBLAST‐treated wounds exhibited more rapid biofilm clearance and wound closure, which agreed with reduced bacterial burden compared to untreated or H_2_O_2_‐treated controls (Figure 7). Although wound closure at Day 12 appeared comparable between µBLAST and H_2_O_2_ treatments, histological analyses revealed marked differences in tissue quality. µBLAST‐treated wounds exhibited more advanced re‐epithelialization and reduced inflammatory responses, indicating improved tissue regeneration beyond simple wound closure. H&E analysis further revealed that µBLAST suppressed pro‐inflammatory activity while enhancing anti‐inflammatory signaling mediated by M2 macrophages and their key effector cytokine, IL‐10. These populations are well known to attenuate inflammatory responses, promote revascularization, and expedite tissue repair, all of which are critical processes for effective skin regeneration [45, 46, 47]. They also prevent chronic inflammation and fibrotic scarring, which were evident in untreated groups. In contrast, H_2_O_2_ treatment led to epidermal thickening with hyperkeratosis, indicative of persistent inflammation and reactive epithelial hyperplasia (Figure 7E) [48]. Abundant granulation tissue was also observed in these wounds, along with excessive adipose tissue accumulation, suggesting exposure of subcutaneous fat and impaired granulation tissue formation. Such adipose occupation of regenerative space may hinder fibroblast activity and angiogenesis, potentially contributing to regeneration failure through sustained inflammation. Overall, these results illustrate that µBLAST functions not only as a physical biofilm‐removal tool but also as an immunomodulatory platform that drives the wound environment toward pro‐regenerative pathways, bridging infection control with tissue regeneration. Histopathological analyses indicate that µBLAST does not cause detectable damage to surrounding tissue under the tested conditions, as evidenced by preserved tissue structure and reduced inflammation. These findings suggest that the localized mechano‐chemical activity of µBLAST is sufficient to disrupt biofilms without causing collateral damage to host tissue. Nevertheless, further studies are required to systematically evaluate long‐term safety, including cytotoxicity and tissue compatibility under repeated treatment conditions.

The sequential combination of µBLAST and gentamicin resulted in further enhanced biofilm eradication, demonstrating superior efficacy compared to antibiotic monotherapy (Figure 8). Consistent with in vitro findings, pretreatment with µBLAST disrupted the biofilm EPS, thereby improving gentamicin penetration into residual bacterial cells and producing a synergistic antibacterial effect that suppressed bacterial burden over time (Figure 8D). Importantly, the log reduction achieved by the combination treatment exceeded that of either µBLAST alone (Figure 7D) or gentamicin monotherapy. In contrast, some wounds treated with gentamicin alone exhibited increased biofilm accumulation, likely due to insufficient antibiotic penetration through the EPS. The moist wound environment associated with the antibiotic ointment may have further stimulated biofilm proliferation (Figure 8B,D) [49]. In addition to biofilm clearance, combination therapy effectively reduced inflammation while promoting anti‐inflammatory responses conductive to normal skin regeneration. Together, these results indicate that sequential treatment with µBLAST and gentamicin synergistically eradicates biofilms and prevents their regrowth in the infected wounds by physically disrupting the biofilm matrix and enhancing antibiotic efficacy.

Recent advances in biofilm eradication strategies have explored diverse approaches, including ultrasound‐based disruption, enzymatic degradation, and bacteriophage therapy, each offering distinct advantages but also important limitations [50, 51, 52, 53, 54, 55]. Ultrasound has demonstrated the ability to disrupt biofilm structures through acoustic cavitation, generating microbubbles that induce mechanical stress and enhance antibiotic penetration. However, its efficacy is often limited in mature viscoelastic biofilms, where dense EPS matrices can reduce penetration and spatial localization of acoustic energy [50, 51]. Enzymatic treatments degrade the EPS matrix, facilitating biofilm dispersal and improving antimicrobial access. While effective in weakening biofilm cohesion, these approaches are often limited by enzyme specificity, stability, and incomplete removal of heterogeneous polymicrobial matrices, particularly in complex in vivo environments [52, 53]. Bacteriophage therapies offer a biologically targeted approach by selectively infecting and lysing bacteria within biofilms. However, their clinical application remains constrained by challenges including host specificity, delivery barriers, limited activity against multispecies biofilms, and emergence of phage resistance [54, 55]. In contrast to these approaches, µBLAST provides a mechanically driven, spatially confined strategy that directly disrupts biofilm structures at the tissue–biofilm interface. By generating bubbles within the biofilm matrix and localizing bubble‐mediated mechanical perturbation, µBLAST enhances mechanical disruption of dense, mature polymicrobial biofilms while simultaneously improving antibiotic efficacy. Unlike enzyme‐ or bacteriophage‐based treatments, which rely on biochemical specificity, µBLAST integrates localized mechano‐chemical activity with enhanced antimicrobial delivery. Nevertheless, µBLAST also has limitations. Its efficacy depends on the availability of H_2_O_2_, MnO_2_‐doped biosilica loading, and catalytic activity. Treatment parameters such as particle dose, H_2_O_2_ concentration, and exposure duration should be optimized to balance biofilm disruption with tissue viability. Additionally, further studies are required to evaluate long‐term safety, scalability, and performance in complex clinical settings.

In summary, this study establishes µBLAST as a multifunctional platform that combines biochemical reactivity with sustained and local mechanical disruption to overcome the refractory resistance of polymicrobial biofilms. By confining MnO_2_‐biosilica beneath H_2_O_2_‐releasing cellulose mesh, µBLAST enhances the penetration of MnO_2_‐biosilica into biofilms, prolong bubble activity within the 3D biofilm matrix, amplify low‐frequency acoustic energy, and concentrates disruptive forces at the biofilm‐wound interface. These effects not only promote efficient biofilm clearance but also synergize with antibiotics to achieve bacterial eradication and suppression at reduced doses, potentially reducing concerns about systemic toxicity and the emergence of antimicrobial resistance. µBLAST also modulated the wound immune environment by promoting M2 macrophage‐associated IL‐10 signaling, thereby coupling infection control with an enhanced pro‐regenerative state. With treatment times limited to 5 min per day, no toxic effects on surrounding tissue were observed. Future studies should optimize mesh design, assess long‐term safety and scalability, evaluate performance in large animal models, and explore integration with diverse therapeutic agents, including growth factors used in tissue regeneration. Together, this work highlights µBLAST as a promising therapeutic modality that bridges infection management with tissue regeneration for large and chronic wounds complicated by biofilm formation [56].

## 4 Experimental Section/Methods

### 4.1 In Vitro Biofilm Culture

For bacteria culture, three colonies of *Pseudomonas aeruginosa* (*P. aeruginosa*, ATCC 15442) and three colonies of methicillin‐resistant *Staphylococcus aureus* (MRSA, ATCC BAA‐1720), each streak‐plated on a Trypticase Soy Agar (TSA) plate (BD BBL) were inoculated into 200 mL of Tryptic Soy Broth (TSB) media (BD Bacto), followed by incubation overnight at 37°C. The concentration of *P. aeruginosa* and MRSA was set to ∼10^7^ and ∼10^8^ CFU/mL, respectively, in which the bacterial suspension had an optical density (OD) of ∼0.2 when measured at a wavelength of 600 nm using a microplate reader (TECAN). For monocultured *P. aeruginosa* or MRSA biofilm, 200 µL of either *P. aeruginosa* or MRSA bacterial suspension was pipetted carefully onto a new TSA plate, forming a disc shape and incubating overnight at 37°C. For co‐cultured polymicrobial biofilm, 10 mL of *P. aeruginosa* bacterial suspension and 10 mL of MRSA bacterial suspension were mixed, and 200 µL of the mixed bacterial suspension was pipetted carefully onto a new TSA plate for biofilm culture. The next day, the disc‐shaped biofilms were punched out from the agar plates using a 20 mm hole punch, rinsed with phosphate‐buffered saline (PBS) to remove planktonic cells, and transferred to a 6‐well plate. Fresh TSB media was added to the disc‐shaped biofilms and incubated overnight. This process was repeated for two more days to cultivate monocultured or co‐cultured biofilms grown for 3 days.

### 4.2 Scanning Electron Microscopy Imaging

Monocultured *P. aeruginosa* or MRSA and co‐cultured polymicrobial biofilm samples were prepared in vitro on TSA plates. For fixation before scanning electron microscopy imaging (SEM), the biofilm samples were immersed in 2% paraformaldehyde and 2.5% glutaraldehyde (both E.M. grade) in 0.1 m Sodium‐cacodylate buffer (pH 7.4) for 4 h, inside the refrigerator. Next, the samples were rinsed with 0.1 m sodium‐cacodylate buffer for 10 min on a shaker table, followed by dehydration in 70% ethanol for 10 min, 90% ethanol for 10 min, and 100% ethanol for 20 min. Then, the biofilm samples were put inside the critical point dryer (Tousimis Autosamdri 931) to fully eliminate moisture. After critical point drying, the biofilm samples were coated with Au/Pd using the sputter coater (Denton Desk‐2 TSC) for 1 min. Finally, the morphologies of the biofilm samples were examined with the SEM (Quanta FEG 450, FEI) at 10 kV.

### 4.3 Confocal Imaging and BiofilmQ Analysis

For immunostaining and fluorescence imaging of in vitro biofilm, fluorescein isothiocyanate (FITC, Sigma‐Aldrich) and concanavalin A tetramethyl rhodamine conjugate (ConA‐TMR, Molecular Probes) were used to label proteins and polysaccharides of the biofilm, respectively. First, to label the nuclei and membranes of the bacterial cells, each disc‐shaped biofilm sample was incubated in 1 mL of 10 mg/mL FITC in 0.1 m NaHCO_3_ buffer for 1 h. Then, to rinse off residual fluorophores, each sample was washed with 5 mL of fresh PBS two times. Next, to label the polysaccharides of EPS, each sample was incubated in 1 mL of 500 µg/mL ConA‐TMR in 0.1 m NaHCO_3_ buffer for 1 h and washed with fresh PBS twice. For fluorescence imaging, 3D z‐stack images of each biofilm sample were captured with an LSM 700 confocal microscope (Zeiss). Then, to analyze the volumes and correlation parameters between EPS and bacterial cells, a comprehensive image cytometry software called BiofilmQ was utilized [28]. 3D renderings of each sample were constructed, and various parameters relating to fluorescent properties were calculated after the confocal images were processed into quantifiable segments using the BiofilmQ software.

### 4.4 Proteomic Analysis

In vitro cultured biofilm was suspended in a lysis buffer of 6 m guanidine (GuHCl), 10 mm tris(2‐carboxyethyl)phosphine (TCEP), 40 mm 2‐chloroacetamide, 0.1% sodium deoxycholate (SDC) in 100 mm tetraethylammonium bromide (TEAB) and then sonicated with a probe sonicator for 2 min. Then, the samples were heated at 95°C for 10 min to disinfect, reduce, and alkylate the samples fully. Samples were centrifuged for 10 min to remove the debris. Supernatants were collected and precipitated with chloroform‐methanol. Dry protein pellets were resuspended in a buffer of 12 mm SDC and 12 mm sodium lauryl sarcosinate in 50 mm TEAB. The protein concentrations were determined by a BCA assay. 50 ug of proteins from the sample were aliquoted and digested with LysC, followed by trypsin. Digested peptides in an SDC‐based buffer were first extracted with ethyl acetate. After acidifying, peptides in the buffer were then desalted with StageTips. Concentrations of the desalted peptides were determined by a BCA colorimetric peptide assay. Then, 200 ng of desalted peptides were analyzed using a Fusion Orbitrap Tribrid mass spectrometer (Thermo Fisher Scientific) coupled to an Ultimate 3000 UHPLC (Thermo Fisher Scientific) over a 60‐minute reversed‐phase gradient. The raw LC‐MS data were searched against the *Pseudomonas aeruginosa* and *Staphylococcus aureus* reference proteome downloaded from UniProt using Mascot v2.8.2, and a reverse decoy database strategy was used to calculate the false discovery rate (FDR).

### 4.5 Rheological Analysis

All rheological tests were performed with a modular compact rheometer (MCR 302, Anton Paar) using sandblasted 20 mm plate geometry. All samples were measured in their double‐layer state, with agar at the bottom and a biofilm formed on top. This method enabled minimizing damage to the biofilm associated with scraping the biofilm off the agar. All samples were tested without trimming to the geometry size for the same reason. The rheology of the agar was tested in the absence of biofilm to allow for inferences to be drawn regarding the biofilm rheology.

Rheological characterization was carried out by performing oscillatory shear tests at an angular frequency of 1 rad/s and fixed temperature of 25°C across a range of strain amplitudes. All tests were carried out with a normal force of 0.5 N to reduce slip and ensure good contact between the biofilm and the geometry. The yield stress and brittility, which describes how abrupt the yielding is, was calculated using the KDR model with Bt. The complete 1‐D version of the model is

(2) $$\sigma +\lambda \left(\dot{\gamma }\right)\dot{\sigma }=\left(\eta_{f}\right)\left(\dot{\gamma }+\frac{\eta_{s}}{G}\ddot{\gamma }\right)$$

where σ is the shear stress, $\dot{\gamma }$ is the shear strain rate and $\ddot{\gamma }$ is its derivative, $\lambda (\dot{\gamma })$ is the rate‐dependent relaxation time, and η_*f*_ is the flow viscosity. The behavior of the biofilm from small to large amplitudes covers the linear and nonlinear regimes and probes all terms of the constitutive relation. The behavior across these amplitudes enables the effective elucidation of the 6 model parameters. The parameter determination and calculation of the model predictions are demonstrated in Figure S3 and Table S1.

The model prediction using KDR with brittility (*Bt*) is calculated using the MATLAB ode15 solver with Equations (2) and (3). The complete model describes the stress‐strain relations under arbitrary deformation and accounts for the abruptness of yielding using the brittility parameter, *Bt* ^2^.

(3) $$\sigma =G\gamma_{rec}+\eta_{s}\overset{.}{\underset{rec}{\gamma }}$$

where $\eta_{f}=\sigma_{y}/\left|{\dot{\gamma }}_{eff}\right|+k|{\dot{\gamma }}_{eff}{|}^{n-1}$, $\lambda \left(\dot{\gamma }\right)=\left(\eta_{f}+\eta_{s}\right)/G$ and $\overset{.}{\underset{eff}{\gamma }}=\overset{.}{\underset{rec}{\gamma }}/Bt+\overset{.}{\underset{unrec}{\gamma }}$.

In Table S1, *G* and η_s_ are recoverable parameters that are defined in the linear regime, σ_y_, k, and *n* are unrecoverable parameters that describe the nonlinear flow regime, represented by the Herchel‐Bulkley model, and *Bt* determines how abruptly the material yields.

All tests were repeated five times with different samples to determine the variability of the biofilm behavior between samples. Variability across all tests from samples of the same batch was low, allowing us to show a single representative case in Figure 2E.

### 4.6 Fabrication of MnO_2_‐Doped Diatom Biosilica

To prepare MnO_2_‐doped diatom biosilica (MnO_2_‐biosilica), 400 mg of diatoms (Diatomaceous Earth‐food grade) were first washed with deionized water and then collected by centrifugation at 1000 rpm for 1 min (Figure S4). After the supernatant was discarded, 40 mL of fresh deionized water and 60 mL of 6 wt.% chitosan solution (Tidal Grow, Tidal Vision) were added to the diatoms, followed by a mixing process for 1 h using a stirrer at 500 rpm (Thermo Fisher Scientific). The chitosan‐coated diatoms were collected by centrifugation at 4000 rpm for 8 min, followed by lyophilization (Labconco) overnight to collect dry powder. Then, 20 mL of 0.1 m KMnO_4_ (Sigma‐Aldrich) was added to 200 mg of chitosan‐coated diatoms and stirred at 500 rpm for 12 h. The MnO_2_‐biosilica were collected by centrifugation at 1000 rpm for 3 min and washed three times with deionized water. Finally, particles were lyophilized overnight and collected as a dry powder for further experiments.

To measure, oxygen gas generation of MnO_2_‐biosilica decomposing H_2_O_2_, O_2_ generation data were collected with an O_2_ gas sensor (Vernier) after mixing 1 mL of 1 mg/mL MnO_2_‐biosilica suspension and 1 mL of 6 wt.% H_2_O_2_ solution. 1 mL of 6% H_2_O_2_ was pipetted into a gas sampling bottle, followed by pipetting 1 mL of 6% H_2_O_2_ to activate O_2_ generation. Then, the O_2_ sensor was positioned on top of the gas sampling bottle to measure the mass of generated O_2_ gas. This was repeated 5 times to generate the average O_2_ generation curve of MnO_2_‐biosilica in 3% H_2_O_2_.

### 4.7 Optical Coherence Tomography Imaging

The spectral‐domain optical coherence tomography (SD‐OCT) imaging system consisted of a custom superluminescent diode (Thorlabs) with a 1,321.6 nm center wavelength and 106.2 nm bandwidth as the light source, the sample arm, including a collimator, a galvanometer scanner, and an objective lens, the reference arm, and a spectrometer consisting of a transmission‐type diffraction grating and a 1024‐pixel InGaAs line‐scan camera (Goodrich). The reflected lights from the sample and reference arms were interfered with in the fiber coupler and detected by a spectrometer. The system provides an axial resolution of around 8 µm and a transverse resolution of around 23 µm. Cross‐sectional images accumulated by 1000 axial scans were acquired at ∼76.5 frames/s. For speckle variance analysis, the intensity of each pixel located at (j, k) in the N‐th image was calculated using Equation (1).

(1) $$I_{SV}=\frac{1}{N}\sum_{i=1}^{N}{\left(I_{ijk}-\frac{1}{N}\sum_{i=1}^{N}I_{ijk}\right)}^{2}$$

where N is number of B‐scans obtained at a single location, and I_ijk_ is the intensity of a pixel with image coordinates (j, k) in the B‐scan indexed by i.

### 4.8 Acoustic Analysis

For all recording experiments, µBLAST was assembled by placing 10 mg of MnO_2_‐biosilica particles beneath the cellulose mesh, and then it was placed on a 2.5 cm × 2.5 cm area of the glass slide. In a standard recording experiment, the acoustic probe (SoundAdvisor 831C, Larson Davis) was placed directly below the sampling area and fixated to the glass slide. 3% H_2_O_2_ was applied to the sample surface, and acoustic data were collected throughout the reaction.

### 4.9 Evaluation of In Vitro Treatment Efficacy of the µBLAST Assembled In Situ

To evaluate the efficacy of treatments in removing polymicrobial biofilms, in vitro agar‐based biofilms were treated with either 3% H_2_O_2_ solution alone or µBLAST. Additionally, to evaluate the role of mesh size and surface energy on the activation of MnO_2_‐biosilica, three mesh types were tested: (i) a nanoporous membrane (Tegaderm), (ii) a cellulose mesh, and (iii) a cellulose mesh coated with polytetrafluoroethylene (PTFE). 2‐ and 4‐day‐old biofilms grown on agar were rinsed gently with PBS to remove planktonic bacteria and transferred to 6‐well plates for treatment. For the treatment, 5 mg of MnO_2_‐biosilica was evenly distributed beneath the membrane and meshes. To initiate oxygen bubble generation, 200 µL of 3% H_2_O_2_ was applied to the surface of the membrane or mesh 3 times at 30‐second intervals. For the condition without any membrane or mesh, 3% H_2_O_2_ was directly applied to the biofilm surface, with or without MnO_2_‐biosilica. After treatment, the samples were transferred to new 6‐well plates and rinsed with PBS to remove residual MnO_2_‐biocilica and surface bubbles. For viability analysis, colony‐forming units (CFU) were quantified post‐treatment. First, the treated samples were placed in 6‐well plates containing 5 mL of fresh PBS and sonicated for 30 min using an ultrasonic cleaner (Fisher Scientific). The resulting suspensions were serially diluted, and 10 µL of each dilution was plated on fresh TSA plates. Following overnight incubation at 37°C, bacterial colonies were counted, and CFU/mL was quantified. For gentamicin treatment only, each 0.1 and 1.0 mg/mL of gentamicin solution was gently dropped on top of the in vitro biofilms and incubated for a day before performing viability analyses. For gentamicin treatment combined with µBLAST, gentamicin solution (Gibco) was dropped on top of in vitro biofilms after pretreatment of µBLAST.

After treatment, confocal laser scanning microscopy images were obtained following the procedures above, and the 3D z‐stack images were inputted in the BiofilmQ software for analysis of cell and matrix volumes as well as the cell‐matrix interactions, visualized by intensity ratio and Manders’ coefficient 3D mapping.

### 4.10 RT‐qPCR Analysis

Total RNA was extracted using TRIzol reagent, and cDNA was synthesized by reverse transcription using iScript cDNA Synthesis Kit (Bio‐Rad). Quantitative PCR was performed using each primer and SYBR Green‐based detection using iTaq Universal SYBR Green Supermix (Bio‐Rad). Relative gene expression levels were normalized to the housekeeping gene (gyrB) and calculated using the 2^‐ΔΔCt^ method, with the control group set to 1.

### 4.11 In Vivo Wound Infection Model

All procedures were conducted according to the guidelines for the care and use of laboratory animals and approved by the Institutional Animal Care and Use Committee (IACUC) of the University of Illinois at Urbana‐Champaign (ID #23153). 8‐week‐old male CD‐1 mice (Charles River Laboratories) were anesthetized with 2% isoflurane in oxygen, and their backs were shaved. Full‐thickness wounds measuring 5 mm in diameter were symmetrically created on both sides of their backs using a disposable biopsy punch (Miltex). Splints were secured around each wound with adhesive and sutures to prevent closure through skin contraction. For the infected wound conditions, 20 µL of a bacterial solution (10^6^ CFU/mL), which included *P. aeruginosa* and MRSA, was applied topically to each wound. The wounds were then covered with a membrane filter to prevent leakage, and Tegaderm (3 m) and bandages were wrapped around them. These dressings were replaced daily.

### 4.12 Treatment of Biofilm‐Infected Wounds

To evaluate the efficacy of the µBLAST in removing biofilm‐infected wounds, we conducted an in vivo study with three treatment groups: (i) untreated control, (ii) 3% H_2_O_2_ only, and (iii) µBLAST. Treatments were initiated on Day 3 following biofilm infection by placing the µBLAST and activating with the 3% H_2_O_2_. Treatments of wounds with either H_2_O_2_ solution or µBLAST were repeated on Days 3, 4, and 5 for a total of three applications. After each treatment, wounds were gently washed with PBS to remove any MnO_2_‐biosilica and biofilm residues.

To assess potential synergy from combining the µBLAST with antibiotics, we designed a second in vivo study with two treatment groups: (i) topical administration of gentamicin only and (ii) sequential treatment with the µBLAST followed by topical gentamicin. In the gentamicin‐only group, 0.1 % gentamicin sulfate ointment (Cosette Pharmaceuticals) was applied to the infected wounds on Days 3, 4, and 5. In the sequential treatment group, the wounds were first treated with the µBLAST for 5 min. The wound was gently rinsed with PBS to remove all residual materials, and gentamicin sulfate ointment was applied on Days 3, 4, and 5.

### 4.13 Evaluation of In Vivo Treatment Efficacy

The bacterial load on the wound was measured using the CFU measurement through serial dilution methodology. Sterile swabs were employed to collect samples from infected wound sites, with the collected material immediately transferred into PBS for suspension. The resulting bacterial suspension was diluted before being cultured on TSA plates. Following 24 h of incubation at 37°C, bacterial colonies were counted for manual enumeration. The final bacterial concentration was determined by applying the appropriate dilution factor to the mean colony count, with results reported as CFU per mL of the original sample.

Wound regeneration quantification was accomplished through taking photographs at 1 to 2‐day intervals over a 14‐day observation period. ImageJ software from the National Institutes of Health facilitated wound area measurements through manual boundary delineation of the residual wound space. Healing progression was expressed as the percentage of tissue regeneration, calculated by determining the ratio of healed tissue area (baseline wound dimension minus current wound size) to the original wound area established at injury initiation. Photographs were acquired under controlled illumination and standardized camera positioning throughout the measurement period.

### 4.14 Histopathological Analysis

The harvested skin tissues on Day 14 were preserved by fixing them in a 4% paraformaldehyde solution to maintain tissue structure. After fixation, the tissues were dehydrated through a graded series of ethanol (70%, 95%, and 100%) and cleared in xylene before being embedded in paraffin. For embedding, the tissues were oriented in molds filled with melted paraffin, which was allowed to solidify on a cold plate. The paraffin blocks were then sectioned into slices that were 5 µm thick using a microtome, and these sections were mounted onto glass slides. After the slides were dried, hematoxylin and eosin staining were performed to analyze tissue morphology. The slides were deparaffinized in xylene, rehydrated through a graded ethanol series (100%, 95%, and 70%), and rinsed with deionized water. The sections were stained with hematoxylin for 3 min, rinsed in deionized water to allow stain development, briefly destained with an acid alcohol solution, and then counterstained with eosin for 30 s. Finally, the slides were dehydrated through graded ethanol, cleared in xylene, and cover‐slipped with a mounting medium to preserve the stained sections.

For the immunofluorescence imaging, the tissue slides were rehydrated through a graded ethanol series and rinsed in deionized water. The rehydrated tissues used the heat‐induced epitope retrieval (HIER) method to expose antigenic sites, enabling antibodies to bind effectively. To prepare the antigen retrieval buffer, a 0.1 m solution of tri‐sodium citrate dihydrate and a 0.1 m solution of citric acid monohydrate were prepared. Specifically, 11.5 mL of the 0.1 m citric acid monohydrate solution was mixed with 88.5 mL of the 0.1 m tri‐sodium citrate dihydrate solution to create a total of 100 mL of 0.1 m citric acid‐sodium citrate buffer at pH 6.0. This buffer was then diluted ten‐fold with deionized water to produce a 0.01 m citric acid‐sodium citrate buffer. Finally, 0.05% Tween‐20 was added to the 0.01 m citric acid‐sodium citrate buffer to prepare the antigen retrieval buffer. The rehydrated tissue slide was immersed in this HIER buffer and processed in a pressure cooker for 10 min. After cooling, the tissue slides were rinsed twice in PBS solution containing 0.05% Tween‐20 (PBS‐Tween), with each rinse lasting 2 min. The tissues were incubated with a blocking buffer containing 5 mg/mL of bovine serum albumin for 30 min. The slides were then placed at 4°C overnight with primary antibodies against IL‐6 (1:100, MA5‐45069, Invitrogen), CD86 (1:200, 14‐0862‐82, Invitrogen), IL‐10 (1:100, MA5‐42656, Invitrogen), and CD206 (1:200, MA5‐16871, Invitrogen). Following this incubation, the tissue slides were treated with secondary antibodies at room temperature for 30 min. Specifically, anti‐mouse AF488 (1:500, A‐11001, Invitrogen) was used for IL‐6, anti‐rat AF488 (1:500, A‐11006, Invitrogen) for CD86, anti‐rabbit AF568 (1:500, A‐11011, Invitrogen) for IL‐10, and anti‐rat AF568 (1:500, A78946, Invitrogen) for CD206. For identification of macrophage polarization, the tissue slides were stained with primary antibodies against F4/80 (1:100, MA5‐16363, Invitrogen) and each CD86 and CD206. Both primary and secondary antibodies were diluted in the blocking buffer. Tissue slides were rinsed in PBS‐Tween several times for 5 min and mounted with 4',6‐Diamido‐2‐phenylindole included mounting medium (00‐4959‐52, Invitrogen). Immunofluorescence images were acquired using an LSM900 confocal microscope (Zeiss).

### 4.15 Statistical Analysis

Statistical data are expressed as means ± standard deviations, and analysis was performed by a one‐way ANOVA with Tukey's post hoc test for pairwise comparisons using Origin 2024b (OriginLab). A p‐value less than 0.05 was considered to indicate statistically significant differences.

## Author Contributions

**Yujin Ahn**: conceptualization, methodology, investigation, visualization, writing – original draft, writing – review and editing. **Joo Hun Lee**: methodology, investigation, visualization, writing – original draft, writing – review and editing. **Jiye Lee**: methodology, visualization. **Christian Hurd**: methodology. **Adam A. Markowicz**: methodology. **Junggeon Park**: methodology, visualization. **Zheyuan Zhang**: investigation. **Simon A. Rogers**: supervision, funding acquisition. **Joanne Hwang**: investigation. **Stephen A. Boppart**: supervision, funding acquisition. **Hyunjoon Kong**: conceptualization, funding acquisition, supervision, writing – review and editing, writing – original draft. **Woonggyu Jung**: supervision. **Guillermo L. Monroy**: investigation.

## Conflicts of Interest

The authors declare no conflicts of interest.

## Supporting information




**Supporting File 1**: advs75999‐sup‐0001‐SuppMat.docx.

**Supporting File 2**: advs75999‐sup‐0002‐MovieS1.mp4.

**Supporting File 3**: advs75999‐sup‐0003‐MovieS2.mp4.

**Supporting File 4**: advs75999‐sup‐0004‐MovieS3.mp4.

**Supporting File 5**: advs75999‐sup‐0005‐MovieS4.mp4.

**Supporting File 6**: advs75999‐sup‐0006‐MovieS5.mp4.

**Supporting File 7**: advs75999‐sup‐0007‐MovieS6.mp4.

## Data Availability

The data that supports the findings of this study are available in the supplementary material of this article.
