# Supplementary material for: Microblasting Wound Dressings Mechanically Disrupt Polymicrobial Biofilms to Enhance Healing in Treatment‐Resistant Wounds
Source: Adv Sci (Weinh). 2026 Jun 10;13(50):e75999. doi: 10.1002/advs.75999 (PMC13336513; doi:10.1002/advs.75999)
Supplement: Supplementary file 1 [file ADVS-13-e75999-s005.docx]

Supporting Information

Microblasting wound dressings mechanically disrupt polymicrobial biofilms to enhance healing in treatment-resistant wounds

Yujin Ahn, Joo Hun Lee, Christian Hurd, Jiye Lee, Junggeon Park, Adam A. Markowicz, Zheyuan Zhang, Joanne Hwang, Guillermo L. Monroy, Simon A. Rogers, Woonggyu Jung, Stephen A. Boppart, and Hyunjoon Kong*

**Figure S1.** Immunofluorescence images of bacterial cells and EPS in monomicrobial and polymicrobial biofilms on Days 2 and 4. [Green: fluorescein isothiocyanate (FITC) for labeling proteins, Red: tetramethyl rhodamine conjugate (ConA-TMR) for labeling polysaccharides].

**Figure S2.** Proteomic analysis of the virulence factors between *P. aeruginosa* monomicrobial biofilms and polymicrobial biofilm co-cultured with *P. aeruginosa* and MRSA. (i) PagL(Lipid A deacylase) relates to LPS (Lipopolysaccharide) immune stimulation regulation, (ii) Elastase relates to host ECM (Extracellular matrix) degradation, inducing host tissue inflammation, (iii) LptH relates to transport LPS to the outer membrane, inducing host tissue inflammation, (iv) LptB is an LPS export system ATP-binding protein, inducing host exposure to LPS, (v) HCP1 relates to inflammatory and cytotoxic effects, inducing immunotoxicity-related inflammation and innate immunity, (vi) Hemolysin relates to cell membrane lysis, inducing damage to tissue and pathogenesis.

**Figure S3.** The model predictions using KDR with brittility (Bt). The complete model describes the stress-strain relations under arbitrary deformation and accounts for the abruptness of yielding using the brittility parameter, Bt^2^.

**Figure S4.** (A) Synthesis process of MnO_2_-doped diatom biosilica (MnO_2_-biosilica), (B) Chitosan is used as a binder of MnO_2_ on the surface of diatom biosilica. KMnO_4_ induces the *in situ* reduction of MnO_4_^-^ to form MnO_2_ nanosheets on the surface of chitosan-diatoms. (C) Mn/Si weight ratio analyzed by EDAX across independently prepared batches. (D) Oxygen generation profiles across independently prepared batches. (E) Cross-sectional OCT images of biofilm after treating with H_2_O_2_ only and MnO_2_-doped biosilica activated by H_2_O_2_. White arrows indicate generated **bubbles**. Over time, the bubbles were observed deeper within the biofilm, suggesting penetration of MnO_2_-doped biosilica particles into the 3D biofilm matrix.

**Figure S5.** Raw OCT images overlaid with speckle variance colormaps after treating (i) unconfined MnO_2_-biosilica and (ii) microblasting wound dressing (μBLAST) for 10 seconds.

**Figure S6.** Raw datasets of power-frequency measurements taken at 5-second intervals for spectrogram analysis. Power at each frequency was recorded during the treatment of (i) unconfined MnO_2_-biosilica and (ii) μBLAST for 60 seconds.

**Figure S7.** Sound intensity of catalytic reaction with single and multiple treatment of H_2_O_2_ solution in unconfined MnO_2_-biosilica and μBLAST. (i) Sound intensity changes after a single treatment of H_2_O_2_ solution for 120 seconds, (ii) Sound intensity changes after multiple treatments (4 times) of H_2_O_2_ solution for 180 seconds.

**Figure S8.** (A) Quantification of bacterial burden in polymicrobial biofilm after each treatment condition. Treatment groups include: (i) diatom biosilica, (ii) MnO_2_ powder, and (iii) MnO_2_-biosilica, (iv) 3% H_2_O_2_ solution, (v) catalase-biosilica treated with H_2_O_2_, and (vi) μBLAST. (B) Quantification of bacterial burden in monomicrobial biofilm after each treatment condition. Treatment groups include: 3% H_2_O_2_ solution, MnO_2_-biosilica activated with H_2_O_2_, and μBLAST activated with H_2_O_2_. Numbers within the plot indicate log reduction relative to the untreated control. Values and error bars represent the average and standard deviation of six samples per condition.

**Figure S9.** Immunofluorescence images of bacterial cells and EPS after treatment with different types of meshes. MnO_2_-biosilica was covered with different meshes, followed by H_2_O_2_ introduction. [Green: fluorescein isothiocyanate (FITC), Red: tetramethyl rhodamine conjugate (ConA-TMR)].

**Figure S10.** (A) Quantification of bacterial burden in polymicrobial biofilm after each treatment condition varied with gentamicin dose. Treatment groups include: (i) H_2_O_2_ pretreatment and (ii) μBLAST pretreatment. (B) Quantification of bacterial burden in monomicrobial biofilm after each treatment condition varied with μBLAST pretreatment and gentamicin dose. Numbers within the plot indicate log reduction relative to the untreated control. N.D. represents non-detectable. Values and error bars represent the average and standard deviation of six samples per condition.

**Figure S11.** Immunofluorescence images of bacterial cells and EPS after treatment with gentamicin monotherapy and a combination of μBLAST pretreatment and gentamicin. [Green: fluorescein isothiocyanate (FITC), Red: tetramethyl rhodamine conjugate (ConA-TMR)].

**Figure S12.** Differential expression of biofilm- and stress-associated genes in *P. aeruginosa* and MRSA following sequential treatment with μBLAST and gentamicin. Values and error bars represent the average and standard deviation of six samples per condition.

**Figure S13.** Immunofluorescence images of wounded tissue from single channels. [Pro-inflammatory factors (CD86, IL-6), Anti-inflammatory factors (CD206, IL-10)].

**Figure S14.** Immunofluorescence images of wounded tissue. [M1 macrophage (F4/80^+^CD86^+^), M2 macrophage (F4/80^+^CD206^+^)].

**Table S1.** The parameter determination and calculation each monomicrobial and polymicrobial biofilms.

|  | Elastic modulus, G (Pa) | Structural viscosity, η_s_ (Pa-s) | Yield stress, σ_y_ (Pa) | Consistency index, k (Pa s^n^) | Exponent, n | Brittility factor, Bt |
| --- | --- | --- | --- | --- | --- | --- |
| *P. aeruginosa* | 11,600 | 600 | 18 | 210 | 0.4 | 7 |
| MRSA | 11,000 | 450 | 18 | 210 | 0.4 | 7 |
| *P. Aeruginosa*  + MRSA | 10,695 | 520 | 50 | 250 | 0.52 | 6 |

**Movie S1.** Application procedure of the μBLAST to the targeted area.

**Movie S2.** Movement of individual MnO_2_-biosilica particles by oxygen bubbles produced during the catalytic reaction.

**Movie S3.** Bubble generation by unconfined MnO_2_-biosilica particles on the substrate surface.

**Movie S4.** Bubble generation by the μBLAST on the substrate surface.

**Movie S5.** Optical coherence tomography of the biofilm treated by H_2_O_2_ alone.

**Movie S6.** Optical coherence tomography of the biofilm treated by MnO_2_-doped biosilica.
